# Differing drivers of decline within a migratory metapopulation has implications for future conservation

**DOI:** 10.1002/ece3.10281

**Published:** 2023-07-14

**Authors:** Luke Ozsanlav‐Harris, Geoff M. Hilton, Larry R. Griffin, Alyn J. Walsh, Lei Cao, Mitch D. Weegman, Stuart Bearhop

**Affiliations:** ^1^ Centre for Ecology and Conservation, College of Life and Environmental Sciences University of Exeter Penryn UK; ^2^ Wildfowl & Wetlands Trust Gloucester UK; ^3^ ECO‐LG Ltd Dumfries UK; ^4^ National Parks and Wildlife Service Wexford Wildfowl Reserve North Slob Ireland; ^5^ State Key Laboratory of Urban and Regional Ecology, Research Centre for Eco‐Environmental Sciences Chinese Academy of Sciences Beijing China; ^6^ Department of Biology University of Saskatchewan Saskatoon Saskatchewan Canada

**Keywords:** Arctic, biologging, climate, long‐distance migrant, nest survival, population decline, remote sensing, white‐fronted goose

## Abstract

Researchers generally ascribe demographic drivers in a single sub‐population and presume they are representative. With this information, practitioners implement blanket conservation measures across metapopulations to reverse declines. However, such approaches may not be appropriate in circumstances where sub‐populations are spatiotemporally segregated and exposed to different environmental variation. The Greenland White‐fronted Goose, *Anser albifrons flavirostris*, is an Arctic‐nesting migrant that largely comprises two sub‐populations (delineated by northerly and southerly breeding areas in west Greenland). The metapopulation has declined since 1999 but this trend is only mirrored in one sub‐population and the causes of this disparity are unclear. Here we compare the drivers and trends of productivity in both sub‐populations using population‐ and individual‐level analysis. We examined how temperature and precipitation influenced population‐level reproductive success over 37 years and whether there was a change in the relationship when metapopulation decline commenced. In addition, we used biologging devices to remotely classify incubation events for 86 bird‐years and modelled how phenology and environmental conditions influenced individual‐level nest survival. Correlations between reproductive success and temperature/precipitation on the breeding grounds have weakened for both sub‐populations. This has resulted in lower reproductive success for the northerly, but not southerly breeding sub‐population, which at the individual‐level appears to be driven by lower nest survival. Earlier breeding ground arrival and less precipitation during incubation increased nest survival in the northerly breeding population, while no factors examined were important for the southerly breeding sub‐population. This suggests reproductive success is driven by different factor(s) in the two sub‐populations. Demographic rates and their environmental drivers differ between the sub‐populations examined here and consequently we encourage further decomposition of demography within metapopulations. This is important for conservation practitioners to consider as bespoke conservation strategies, targeting different limiting factors, may be required for different sub‐populations.

## Graphical abstract

Greenland White‐fronted Geese exhibit a metapopulation structure. The two largest sub‐populations show different trends in reproductive success and respond to enviromental factors differently. They therefore may require different conservation strategies to reverse declines. This highlights that more holisitc approaches to the conservation and monitoring of other migrant metapopulations may be required.

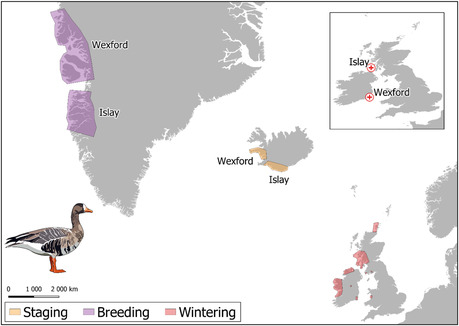

## 1 INTRODUCTION

In order to diagnose population decline and design appropriate mitigation strategies, it is vital to accurately estimate demographic rates such as survival and reproductive success, and identify rate‐limiting environmental factors. Demographic data are often collected from a small portion of a species' range and estimates are presumed to be representative of the entire range. However, many populations exhibit a metapopulation structure with discrete sub‐populations, linked via emigration/immigration, having different demography (Dugger et al., 2010; Hewson et al., 2016; Morrison et al., 2016). Therefore study of several sub‐populations is required for inference about the mechanisms driving metapopulation structure and change.

Understanding how the demography of a metapopulation is influenced by environmental conditions and the contribution of each individual sub‐population to the overall metapopulation trend can be difficult for a number of reasons, including: (1) multiple interacting environmental conditions, which could differ between sub‐populations, can be hard to disentangle and their effects on demography may be additive, subtractive or multiplicative (Amano et al., 2020; Dugger et al., 2010); (2) carry‐over effects from previously encountered environmental conditions (Inger et al., 2010; Norris et al., 2004) as well as direct effects of current conditions (Alves et al., 2019; Brawn et al., 2017) can influence demography; and (3) individual sub‐populations may contribute disproportionately to the demography of the overall metapopulation and demographic changes in one sub‐population may affect trends in other sub‐populations, especially for ‘sink’ sub‐populations that are subsidised by immigration from ‘source’ sub‐populations. It is rarely feasible to examine all past and present environmental conditions across a number of sub‐populations, but missing data on relatively large sub‐populations can impair our ability to fully understand metapopulation dynamics.

In migratory species, understanding metapopulation dynamics is further complicated by seasonal use of multiple sites throughout the annual cycle, which could differentially contribute to metapopulation trends. Groups of individuals that are spatially separated during one period of the annual cycle may co‐occur during other periods, which creates a challenge for practitioners wanting to estimate the demography and conduct hypothesis tests of drivers for specific sub‐population trajectories. Where sub‐populations are spatially separated, they will likely experience different environmental conditions which can differentially influence current (Alves et al., 2019; Brawn et al., 2017) and future demography via carry‐over effects (Inger et al., 2010; Norris et al., 2004). Moreover, larger magnitudes of spatiotemporal segregation between sub‐populations will increase the likelihood of environmental separation and in turn differences in demography (Acker et al., 2021). Species for which we can readily identify and monitor distinct sub‐populations across the full annual cycle provide unique opportunities to understand the influences of environmental conditions across metapopulation units.

**FIGURE 1:**
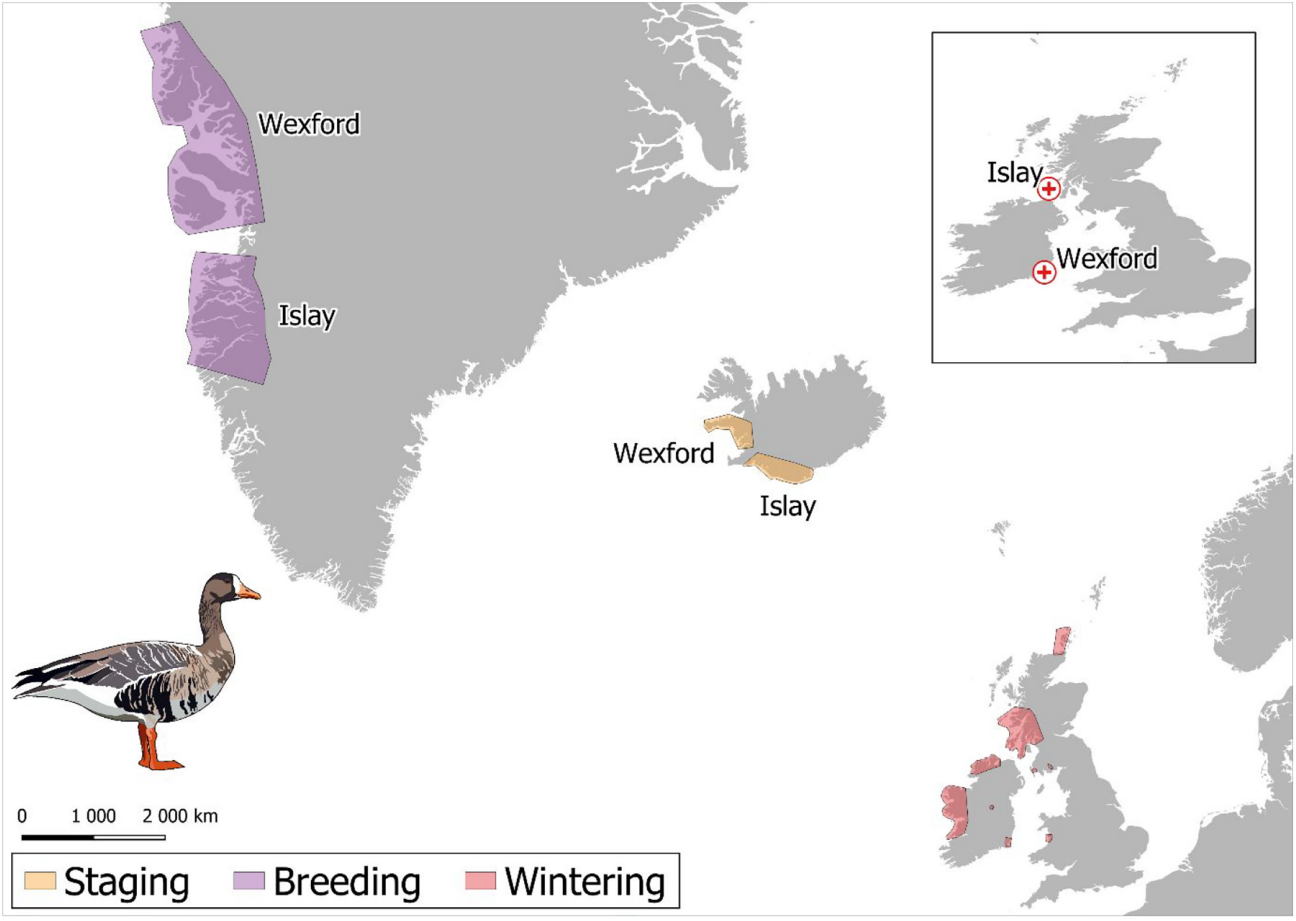
The global range of GWfG during breeding, staging and non‐breeding. The Wexford and Islay sub‐populations' ranges throughout the annual cycle are delineated.

The Greenland White‐fronted Goose (GWfG hereafter) is a long‐distance migrant that winters at c70 discrete sites in Britain and Ireland, stages in Iceland and breeds in west Greenland (Figure 1). It provides a tractable system to understand metapopulation dynamics as marking, frequent resighting and GPS tagging is feasible, allowing sub‐populations to be delineated throughout the annual cycle. While the overwinter metapopulation structure is complex, we focus on two sub‐populations wintering in Islay, Scotland and Wexford, Ireland that hold the majority of individuals (>70%) and use broadly separate regions throughout the annual cycle. Individual marking showed that 85% of birds at Wexford 1 year, return the following year (Wilson et al., 1991) and only 4% of birds switch spring staging sites between years (Fox et al., 2002). Although there is evidently some exchange, overall birds wintering at Wexford have a more northerly breeding distribution and more westerly staging distribution compared to the Islay sub‐population (Figure 1, Figure S1) with all GPS‐tracked individuals showing high nest site fidelity (Figure S2).

**FIGURE 2:**
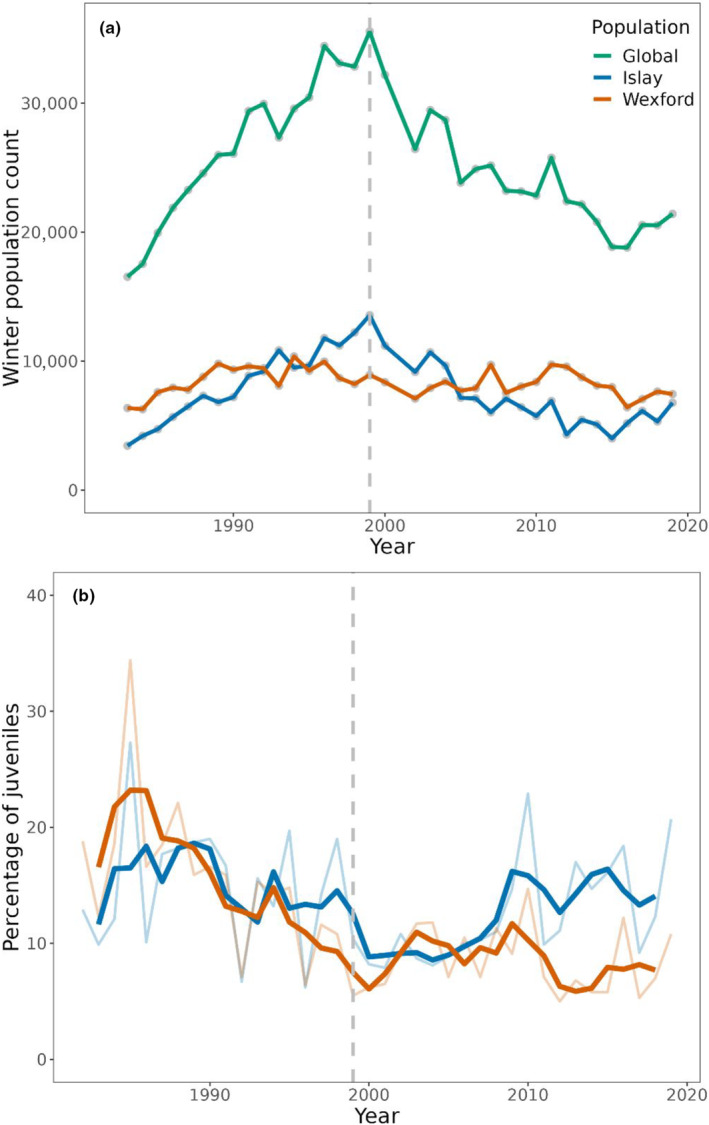
Population and demographic trends of GWfG from 1983 to 2019. (a) The metapopulation count and the Islay, Scotland & Wexford, Ireland sub‐population counts (data from annual March census). (b) The percentage of young observed in the Islay and Wexford sub‐populations (paler thinner lines = observed data; darker line = 3‐year running average). The grey dashed line marks the metapopulation peak in 1999.

The GWfG metapopulation has declined by ~50% since 1999 (Figure 2a), driven by changes in productivity and adult survival (Figure 2b) (Weegman et al., 2021). While the metapopulation trend is an important monitoring metric for conservation and management, it is not mirrored by both sub‐populations. The Islay sub‐population matches the recent metapopulation decline, whereas the Wexford sub‐population has remained relatively constant (Figure 2a). Sub‐population stability at Wexford has been achieved through high net immigration, which has offset low reproductive output (Weegman et al., 2016). Comparatively less is known about the demography of the Islay sub‐population, but reproductive success has been higher than Wexford birds since 2008, although overall numbers have declined in this period. These contrasting trends highlight that a single focal sub‐population might not be a reliable indicator of overall metapopulation demography, creating a need for greater focus on sub‐populations to holistically understand the causes and consequences of metapopulation change.

Here we focus on reproductive success as it is an important driver of the overall metapopulation trajectory in GWfG, yet differs at the sub‐population level (Weegman et al., 2021). There is also mounting evidence that climate change is decreasing reproductive success of Arctic migratory birds, particularly shorebirds (Kubelka et al., 2022). Since GWfG breed in remote Arctic regions, it can be difficult to understand drivers of reproductive success by searching for territorial pairs with teams on the ground, yet such study is critical for developing conservation strategies that improve reproductive success. In GWfG, population‐level reproductive success is estimated annually as a range‐wide age‐ratio survey within wintering areas (referred to as productivity hereafter). This survey provides the proportion of juveniles within wintering areas and therefore encompasses both the number of chicks fledged and juvenile mortality between fledging and arrival at the wintering areas. This makes it difficult to identify the point at which breeding fails and thus identify contributing factors. Biologging devices provide a unique opportunity to remotely assess productivity at the individual‐level much earlier in the reproductive period, for example, nesting success (Ozsanlav‐Harris et al., 2022). Using a set of known tracked breeders it is possible to derive movement metrics from GPS data and energy expenditure from accelerometer (Acc) data (Qasem et al., 2012) to determine patterns indicative of incubation. A rule‐based classification system can then be used to classify days as ‘incubation’ or ‘not’ using a method that could be applied to other species with uniparental incubation (Ozsanlav‐Harris et al., 2022).

In this study, we used biologging devices to remotely classify individual‐level reproductive life histories in combination with surveyed population‐level productivity. At the individual‐level we tested whether environmental conditions on the breeding grounds and phenology have driven breeding deferral, success and nest survival and whether the drivers of nest survival differed between sub‐populations. At the population‐level we tested whether environmental conditions on the breeding grounds drive sub‐population productivity and whether the drivers differed between sub‐populations over time. Combining population‐ and individual‐level approaches allowed us to understand the environmental drivers of reproductive success within a metapopulation and importantly build on previous work, for example Hewson et al. (2016) and Morrison et al. (2016), by relating changes in sub‐population‐specific demographic rates to individual‐level life histories, similar to Buechley et al. (2021).

## 2 MATERIALS AND METHODS

### 2.1 Individual‐level methods

#### 2.1.1 Device deployment

Geese were caught at baited sites using cannon nets in Scotland (34 birds), Ireland (28 birds) and Iceland (nine birds) during February 2017–December 2021. All tagging was performed under licence from the British Trust for Ornithology (permit no: /A/5436). Geese were sexed, measured, weighed and aged post‐capture and only devices deployed on adult females were used in this study. Where sexing was ambiguous, a device was not deployed. Ornitela Global System for Mobile Communication (GSM) (model N38; 38 g) devices were used in this study. These tags collected 1 GPS location every 15 min and a 3 s Acc burst every 6 min at 10 Hz. Each individual was tracked for 0–4 breeding seasons, providing the following number of tracked breeding seasons across individuals: 1 year (23), 2 years (13), 3 years (8) and 4 years (3). Tracked breeding seasons required data from May, June and July. Birds spending the winter in Wexford and/or staging in Hvanneryi, Iceland were assigned to the Wexford sub‐population and those wintering in Scotland and the north of Ireland were assigned to the Islay sub‐population, as these two groups used the same regions in Iceland and Greenland. Morphology was compared between the two sub‐populations using linear models (*n* = 67 individuals, see Figure S3) and there were no significant differences in body mass, wing length or scaled mass index (calculated from body mass and head‐bill length using the method in Peig & Green, 2009).

#### 2.1.2 Classification of incubation

We classified incubation events from biologging data using the methodology described in Ozsanlav‐Harris et al. (2022). This methodology uses biologging devices to measure movement from GPS data and energy expenditure from Acc data (Qasem et al., 2012). Using a set of known breeders (*n* = 9 bird‐years) it was possible to determine movement and energy expenditure patterns indicative of incubation. A rule‐based classification system was then used to classify days as ‘incubation’ or ‘not’ for individuals with unknown breeding success. When performing leave‐one‐out cross‐validation, a successful incubation was classified for each of the nine known successful breeders in the training set. In addition, biologging data from four males and one immature female, which are known not to incubate (Stroud, 1982), were not classified with a single day of incubation. Only breeding seasons with a GPS fix frequency <60 min and an Acc burst frequency <24 min were included because coarser resolutions reduced incubation classification accuracy (Ozsanlav‐Harris et al., 2022).

#### 2.1.3 Environmental conditions and migratory phenology

We annotated individual movement paths with the following environmental variables that have previously been found to influence reproductive success in Arctic breeding geese: precipitation (Lecomte et al., 2009), temperature (Doyle et al., 2020), Normalized Difference Vegetation Index (NDVI) (Doiron et al., 2015) and percentage snow cover (Nolet et al., 2020). The Movebank Env‐DATA system (Kays et al., 2013) was used to annotate the following European Centre for Medium‐Range Weather Forecasts (ECMWF) products onto tracks using bilinear interpolation: ECMWF Interim Full Daily SFC Temperature (2 m above ground) and ECMWF Interim Full Daily SFC‐FC Total Precipitation. The following data products from the Moderate Resolution Imaging Spectroradiometer (MODIS) satellite were annotated onto tracks using inverse‐weighted interpolation: MODIS Snow 500 m Daily Terra NDSI Snow Cover and MODIS Land Vegetation Indices 500 m 16 days Terra NDVI. Due to slight differences in GPS sampling schedules across tags, we resampled tracks prior to annotation. This prevented bias in the average environmental conditions along each track. For temperature and precipitation we annotated values onto the 08:00, 12:00 and 16:00 UTC fixes, whereas NDVI and snow cover were annotated onto only the 12:00 UTC fix, as these variables change over longer temporal scales.

Arrival environmental conditions were calculated for the first 10 days after an individual arrived on the breeding grounds (see classification of arrival date below). NDVI, snow cover and temperature were averaged over the 10 days and precipitation values were summed. The 10‐day period was chosen as it is key time to replenish depleted body reserves after migration (Fox & Bergersen, 2005) and acquire resources for breeding (Lameris et al., 2017) (a 20‐day window was also trialled but, similar to the 10‐day window, this variable was not considered an important driver of breeding). Temperature, NDVI and percentage snow cover (but not precipitation) were highly correlated within the 10‐day period so we ran a principle component analysis (PCA) with these three variables. The first component axis explained 75.3% of the total variance (PCA loadings in Figure S4). The first principal component axis (Climatic PCA axis 1 hereafter) was used for modelling and high positive values represented warm temperatures, low snow cover and high NDVI. To assess if the climate during incubation was associated with nest failure, we set up right‐centred rolling windows during incubation that averaged temperature and summed precipitation. These rolling windows ranged in size from 1 to 10 days on the basis that nest failure may be caused by short‐term bouts of severe weather, or by a sustained period of inclement weather.

The absolute displacement method in Soriano‐Redondo et al. (2020) was used to calculate Greenland arrival dates for each individual. This method identifies an ‘unmistakable’ migratory period, in our instance the North Atlantic sea crossing. This period is then extended forward and backwards in time (giving arrival and departure dates, respectively) as long as the individual travels more than 50 km within a day or between consecutive days. Breeding arrival date was used in subsequent models as it often correlated with higher breeding success (Morrison et al., 2019). The repeatability of breeding arrival dates was measured for individuals tracked over 2+ years using the *rptR* R package (Stoffel et al., 2017) (*n* = 101 bird‐years, additional tags were used that did not meet data requirements for incubation classification; Table S1). Breeding site arrival date, mean centred by year to control for between‐year variability was the response variable and individual ID was fit as a random intercept term. To evaluate uncertainty in the repeatability estimate, we ran 1000 parametric bootstraps.

#### 2.1.4 Modelling variation in incubation initiation and success

To model the probability of individuals commencing incubation we used generalized mixed models described mathematically in Equations (1), (2), (3) (*n* = 85 bird years). It was not possible to distinguish commencement of a breeding attempt without incubation, for example preparing a nest site and perhaps initiating laying, from birds that tactically deferred breeding activities. $Y_{\mathrm{iy}}$ was the binary response variable indicating whether individual *i*, in year *y* commenced incubation (1 for commenced; 0 otherwise) and $p_{\mathrm{iy}}$ was the probability individual *i* commenced incubation in year *y*. Firstly, we modelled whether environmental variables and phenology influenced incubation initiation across sub‐populations (Equations 1 and 2), fitting the following fixed effects: (1) total precipitation in the 10 days after Greenland arrival $\left({\text{ArPr}}_{\mathrm{iy}}\right)$; (2) Climatic PCA axis 1 $\left({\text{Clim}}_{\mathrm{iy}}\right)$; (3) Greenland arrival date, mean centred by year $\left({\mathrm{Ar}}_{\mathrm{iy}}\right)$; and (4) year as a category $\left({\mathrm{Yr}}_{y}\right)$. Second, we modelled whether probability of incubation initiation differed between sub‐populations (Equations 1 and 3) since environmental variables differed between the two sub‐populations. The following fixed effects were fit: (1) sub‐population $\left({\mathrm{Pop}}_{i}\right)$; and year as a category $\left({\mathrm{Yr}}_{y}\right)$. Individual *i* was included as a random intercept ($\varepsilon_{i}$) for both models.

(1) $$Y_{\mathrm{iy}}∼\text{Bernoulli}\left(p_{\mathrm{iy}}\right)$$

(2) $$\text{logit}\left(p_{\mathrm{iy}}\right)=\beta_{0}+\beta_{1}{\text{ArPr}}_{\mathrm{iy}}+\beta_{2}{\text{Clim}}_{\mathrm{iy}}+\beta_{3}{\mathrm{Ar}}_{\mathrm{iy}}+\beta_{4}{\mathrm{Yr}}_{y}+\varepsilon_{i}$$

(3) $$\text{logit}\left(p_{\mathrm{iy}}\right)=\beta_{0}+\beta_{1}{\mathrm{Pop}}_{i}+\beta_{2}{\mathrm{Yr}}_{y}+\varepsilon_{i}$$

To discern between nests with eggs that hatched and those that did not, we used a 25‐day inclusive cut‐off to define a successful incubation, based on historic observations on the breeding grounds (Stroud, 1982). Generalized mixed models, described mathematically in Equations (4), (5), (6), were used to estimate the probability of successful incubation ($S_{\mathrm{iy}}$) for individual *i* in year *y* (*n* = 85 bird‐years). $Z_{\mathrm{iy}}$ was the binary response variable indicating if individual *i*, in year *y* successfully incubated. First, we modelled whether environmental variables and phenology influenced incubation success across sub‐populations (Equations 4 and 5), fitting the following fixed effects: (1) total precipitation in the 10 days after Greenland arrival $\left({\text{ArPr}}_{\mathrm{iy}}\right)$; (2) Climatic PCA axis 1 $\left({\text{Clim}}_{\mathrm{iy}}\right)$; (3) Greenland arrival date, mean centred by year $\left({\mathrm{Ar}}_{\mathrm{iy}}\right)$; and (4) year as a category $\left({\mathrm{Yr}}_{y}\right)$. Second, we modelled whether incubation success differed between sub‐populations (Equations 4 and 6) since environmental variables differed between the two sub‐populations. The following fixed effects were fit: (1) sub‐population $\left({\mathrm{Pop}}_{i}\right)$; and year as a category $\left({\mathrm{Yr}}_{y}\right)$. Individual *i* was included as a random intercept ($\varepsilon_{i}$) for both models.

(4) $$Z_{\mathrm{iy}}\sim \text{Bernoulli}\left(S_{\mathrm{iy}}\right)$$

(5) $$\text{logit}\left(S_{\mathrm{iy}}\right)=\beta_{0}+\beta_{1}{\text{ArPr}}_{\mathrm{iy}}+\beta_{2}{\text{Clim}}_{\mathrm{iy}}+\beta_{3}{\mathrm{Ar}}_{\mathrm{iy}}+\beta_{4}{\mathrm{Yr}}_{y}+\varepsilon_{i}$$

(6) $$\text{logit}\left(S_{\mathrm{iy}}\right)=\beta_{0}+\beta_{1}{\mathrm{Pop}}_{i}+\beta_{2}{\mathrm{Yr}}_{y}+\varepsilon_{i}$$

Equations 2, 3, 5 and 6 were checked for violations of the underlying distributional assumptions using the *DHARMa* package (Hartig, 2021) and 95% confidence intervals calculated for all parameters.

#### 2.1.5 Modelling variation in nest survival

To assess the effect of environmental conditions and migratory phenology on survival of initiated nests, we used a mixed effects Cox proportional hazards model (Equation 7). A nest was deemed successful if incubation occurred for at least 25 days (Stroud, 1982) and failed if incubation ceased prior to this. These models describe the hazard of incubation failure occurring over time $\lambda \left(t\;|\;Z_{\mathrm{iy}}\left(t\right)\right)$, which gives the hazard ratio at time point *t*, given a set of covariates *Z* whose values can be updated based on time *t*. This can be expressed as a function of the baseline probability or hazard rate, $\lambda_{0}\left(t\right)$, which can be modified via a series of time‐constant or ‐varying covariates. In our case covariates that described migratory phenology and environmental conditions prior to incubation were time‐constant, being fixed prior to incubation initiation. We also used time‐varying environmental covariates whose values changed throughout incubation. For instance, ${\mathrm{Pr}}_{\mathrm{iy}}\left(t\right)$ represents the total daily precipitation for individual *i*, in year *y*, at time point *t*. The time points *t* in our analysis are consecutive days.

We carried out separate analysis for the Wexford (*n* = 26 bird‐years) and Islay (*n* = 40 bird‐years) sub‐population as we believed that drivers of nest survival might differ between the sub‐populations. Interacting a sub‐population term with each climatic and phenological variable in a single model would have led to over parameterisation. For each sub‐population, we fit the following time‐constant fixed effects: (1) total precipitation in the 10 days after Greenland arrival $\left({\text{ArPr}}_{\mathrm{iy}}\right)$; (2) Climatic PCA axis 1 $\left({\text{Clim}}_{\mathrm{iy}}\right)$; (3) Greenland arrival date, mean centred by year $\left({\mathrm{Ar}}_{\mathrm{iy}}\right)$; and (4) year as a category $\left({\mathrm{Yr}}_{y}\right)$. We fit the following time‐varying fixed effects: (1) total precipitation $\left({\mathrm{Pr}}_{\mathrm{iy}}\left(t\right)\right)$; and (2) average temperature $\left({\text{Temp}}_{\mathrm{iy}}\left(t\right)\right)$. Individual, *i*, was fit as a random intercept term $\left(\varepsilon_{i}\right)$.

(7) $$\lambda \left(t\;|\;Z_{\mathrm{iy}}\left(t\right)\right)=\lambda_{0}\left(t\right)\exp\left(\beta_{1}{\text{ArPr}}_{\mathrm{iy}}+\beta_{2}{\text{Clim}}_{\mathrm{iy}}+\beta_{3}{\mathrm{Ar}}_{\mathrm{iy}}+\beta_{4}{\mathrm{Yr}}_{y}+\beta_{5}{\mathrm{Pr}}_{\mathrm{iy}}\left(t\right)+\beta_{6}{\text{Temp}}_{\mathrm{iy}}\left(t\right)+\varepsilon_{i}\right)$$

We ran a series of models for each sub‐population that allowed total precipitation $\left({\mathrm{Pr}}_{\mathrm{iy}}\left(t\right)\right)$ and average temperature $\left({\text{Temp}}_{\mathrm{iy}}\left(t\right)\right)$ to be calculated over window sizes of 1, 2, 3, 4, 5 and 10 days and ranked them by AICc (relative estimate of model prediction error for small sample sizes), reporting the confidence intervals from the model with the lowest AICc. This allowed for a series of wet or cold days to cause nest failure. Temperature and precipitation were chosen to be time‐varying as they are available from National Centers for Environmental Prediction (NCEP) at a daily resolution without missing values. Finally, to test whether nest survival differed between sub‐populations, we ran an additional Cox proportional hazards model with all bird‐years from both sub‐populations (*n* = 66 bird‐years). This model contained sub‐population and year as time‐constant fixed effects and individual *i* as a random intercept term. The *survival* package (Therneau, 2021) was used for analysis and the proportional hazards assumption was tested using the *survminer* package (Kassambara et al., 2020).

### 2.2 Population‐level methods

#### 2.2.1 Productivity counts

Annual counts of juveniles and adults in wintering flocks have been conducted since 1983. Juveniles are visually separable from adult birds owing to distinctive plumage characteristics (Weegman et al., 2021). We use counts for the Islay, Scotland and Wexford, Ireland sub‐populations in further analysis.

#### 2.2.2 Temporal trends in productivity

To test for temporal trends in productivity for both sub‐populations, we used generalized least squares regression via the *gls()* function in the *nlme* R package (Pinheiro et al., 2022). ${\mathrm{Juv}}_{\mathrm{yp}}$ was the response variable, representing the number of juveniles per 1000 birds in year *y*, for sub‐population *p* (Equation 8). For this model we fit an interaction between two fixed effects: (1) year $\left({\mathrm{Yr}}_{y}\right)$, continuous variable; and (2) sub‐population $\left({\mathrm{Pop}}_{p}\right)$.

(8) $${\mathrm{Juv}}_{\mathrm{yp}}=\beta_{0}+\beta_{1}{\mathrm{Yr}}_{y}+\beta_{2}{\mathrm{Pop}}_{p}+\beta_{3}{\mathrm{Yr}}_{y}\times {\mathrm{Pop}}_{p}$$

Residuals were significantly auto‐correlated for the null (intercept‐only) model (Durbin‐Watson test, *p* = .001) but not for the maximal model in Equation 8 (Durbin‐Watson test, *p* = .195). To be conservative we fit a first‐order autoregressive covariance structure to year $\left({\mathrm{Yr}}_{y}\right)$ using the *corAR1()* function in the correlation argument of *gls()*. We trialled a second‐order autoregressive structure but this increased model AICc.

#### 2.2.3 Environmental conditions

**FIGURE 3:**
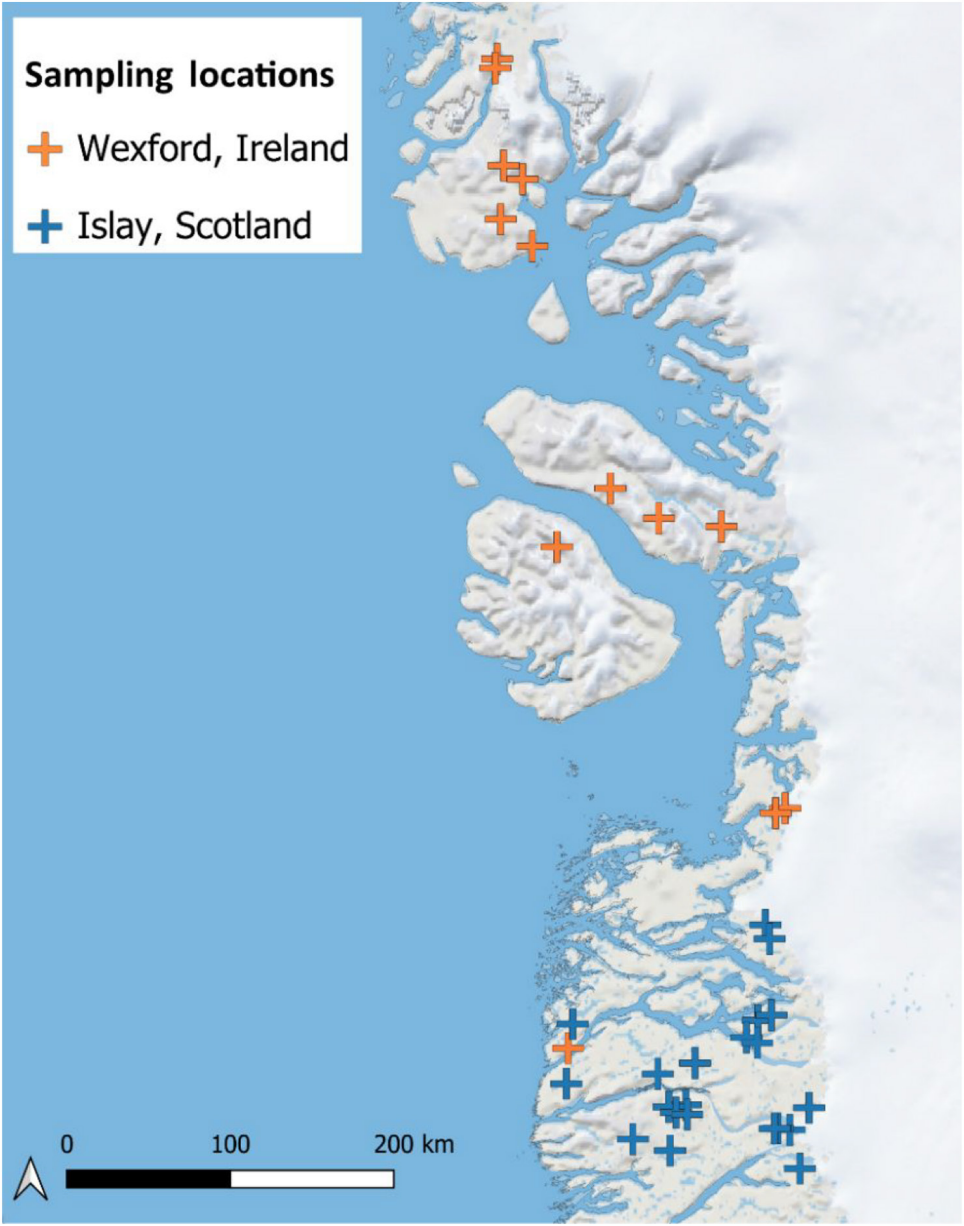
Map of tracked breeding locations from the Wexford and Islay sub‐populations used to sample annual climatic conditions.

We used tracking data from individuals that wintered in Islay, Scotland and Wexford, Ireland to calculate breeding ground environmental conditions for both sub‐populations. We calculated the median values of longitude and latitude during incubation for each tracked individual. As individuals tend to be faithful to breeding sites, we randomly selected a single breeding location for individuals tracked in multiple years. This gave 13 locations from the Wexford sub‐population and 24 locations from the Islay sub‐population. These 37 locations (Figure 3) cover the known GWfG breeding range and were used as sampling points to calculate representative environmental conditions (temperature and precipitation) for both sub‐populations through time.

Temperature and precipitation time series were created from 1 April to 31 August every 6 h for the years 1983–2019. This date range covers the full breeding season (nesting and chick growth) and the month prior, which can effect conditions upon arrival (Boyd & Fox, 2008). Temperature and precipitation were annotated at each of the 37 locations for each time step in the time series using the Movebank Env‐DATA system (Kays et al., 2013). The following ECMWF data products were annotated using bilinear interpolation: ECMWF Interim Full Daily SFC Temperature (2 m above ground) and ECMWF Interim Full Daily SFC‐FC Total Precipitation. From the annotated time series we calculate the mean number of days for which average temperature was below 0°C and the total precipitation over three time periods for both sub‐populations each year. The time periods were the entire breeding period (1 April–31 August), pre‐hatching period (1 April–20 June) and post‐hatching period (20 June–31 August). June 20th was used since it was the median hatch date from our individual‐level analysis. We chose to use days below 0°C instead of average temperature because we believe it more accurately reflects resource access since below 0°C soils become frozen and precipitation is more likely to fall as snow.

#### 2.2.4 Modelling variation in productivity

We modelled variation in GWfG productivity, measured as the number of juveniles per 1000 birds in winter flocks, using generalized least squares regression via the *gls()* function. We modelled the Islay and Wexford sub‐populations separately using Equation 9 (*n* = 37 years). To test whether environmental conditions over different time periods explained different amounts of variation in productivity, we fit 10 models (including a null model) for each sub‐population. The number of days below 0°C and total precipitation in each model were calculated over different combinations of the entire breeding season, pre‐hatching period or post‐hatching period (Table 1). For each model, ${\mathrm{Juv}}_{y}$ represents the number of juveniles per 1000 birds in year *y*. We fit the following variables as fixed effects: (1) the metapopulation trend as a two‐level factor, ‘increasing’ prior to 1999 and ‘decreasing’ after $\left({\mathrm{Tr}}_{y}\right)$; (2) the total precipitation during the time period of interest $\left({\mathrm{Pr}}_{y}\right)$; and (3) the number of days below 0°C for the time period of interest $\left({\text{Freeze}}_{y}\right)$. ${\mathrm{Pr}}_{y}$ and ${\text{Freeze}}_{y}$ were interacted with ${\mathrm{Tr}}_{y}$ to allow slope estimates to differ between sub‐populations. The residuals for two models fitted to the Islay data and seven fitted to the Wexford data were significantly auto‐correlated. Therefore for all models we fit a first‐order autoregressive covariance structure to year using the *corAR1()* function in the correlation argument of *gls()* (Figures S5 and S6). We trialled a second‐order autoregressive covariance structure but this increased model AICc.

(9) $${\mathrm{Juv}}_{y}=\beta_{0}+\beta_{1}{\mathrm{Tr}}_{y}+\beta_{2}{\text{Freeze}}_{y}+\beta_{3}{\mathrm{Pr}}_{y}+\beta_{4}{\text{Freeze}}_{y}\times {\mathrm{Tr}}_{y}+\beta_{5}{\mathrm{Pr}}_{y}\times {\mathrm{Tr}}_{y}$$

**TABLE 1:** Model selection table to identify the climatic conditons, during different time periods, that explain the most variation in productivity of the Islay and Wexford sub‐populations.

Response variable	Entire *Precip*	Entire *Freeze*	Pre‐hatch *Precip*	Pre‐hatch *Freeze*	Post‐hatch *Precip*	Post‐hatch *Freeze*	AICc	Δ AICc
*Juv*_*Wexford*_	‐	‐	X	X	‐	‐	370.96	0
*Juv*_*Wexford*_	X	‐	‐	X	‐	‐	374.09	3.13
*Juv*_*Wexford*_	‐	‐	‐	X	‐	X	376.05	5.09
*Juv*_*Wexford*_	‐	X	X	‐	‐	‐	376.86	5.90
*Juv*_*Wexford*_	X	X	‐	‐	‐	‐	378.82	7.86
*Juv*_*Wexford*_	‐	X	‐	‐	X	‐	380.86	9.90
*Juv*_*Wexford*_	‐	‐	‐	‐	‐	‐	401.56	30.60
*Juv*_*Wexford*_	‐	‐	‐	‐	X	X	406.08	35.12
*Juv*_*Wexford*_	X	‐	‐	‐	‐	X	406.61	35.65
*Juv*_*Wexford*_	‐	‐	‐	‐	X	X	406.92	35.96
*Juv*_*Islay*_	X	X	‐	‐	‐	‐	388.10	0
*Juv*_*Islay*_	‐	X	X	‐	‐	‐	389.30	1.20
*Juv*_*Islay*_	X	‐	‐	X	‐	‐	389.74	1.63
*Juv*_*Islay*_	‐	X	‐	‐	X	‐	390.83	2.73
*Juv*_*Islay*_	‐	‐	X	X	‐	‐	392.63	4.53
*Juv*_*Islay*_	‐	‐	‐	X	X	‐	392.75	4.65
*Juv*_*Islay*_	‐	‐	‐		X	X	392.87	4.76
*Juv*_*Islay*_	X	‐	‐	‐	‐	X	392.96	4.86
*Juv*_*Islay*_	‐	‐	‐	‐	‐	‐	401.56	13.46
*Juv*_*Islay*_	‐	‐	‐	X	X	‐	402.76	14.66

*Note*: Models were run separately for each sub‐population and ranked by AICc. ‘Entire’: entire breeding period (1 April–31 August), ‘Pre‐hatch’: pre‐hatching period (1 April–20 June), ‘Post‐hatch’: post‐hatching period (20 June–31 August).

For each sub‐population we ranked the 10 models using AICc and retained the model with the lowest AICc for inference. To test for temporal trends in ‘total precipitation’ and ‘number of days below 0°C’ from the highest ranked model for each sub‐population, we used generalized least squared regression with year as a continuous fixed effect and a first‐order autoregressive covariance structure to year.

### 2.3 General statistical procedures

The population‐level explanatory variables were not z‐transformed prior to modelling in order to aid understanding of the effect sizes on the true data scale. For the individual‐level analysis all explanatory variables were z‐transformed prior to modelling to allow comparison of effect sizes within regression models and to prevent scaling issues in parameter estimation. All analysis was carried out using R v3.6.3 (R Core Team, 2020). And for data manipulation we used the *tidyverse* (Wickham et al., 2019) and *data.table* (Dowle & Srinivasan, 2019) packages.

## 3 RESULTS

### 3.1 Individual‐level results

#### 3.1.1 Migratory phenology

Individual breeding ground arrival dates were moderately repeatable (*β* = 0.44 [CI: 0.21, 0.63]) within individuals (Figure S7). Suggesting individuals that arrive early on the breeding grounds in a given year (relative to the population) tended to arrive early in other years.

#### 3.1.2 Classification of incubation events

In total, evidence of incubation was assessed for 85 bird‐years that had GPS and Acc data for May, June and July. Among these, there were 19 bird‐years in which no incubation was identified, 45 bird‐years in which incubation started but failed and 21 bird‐years in which incubation proceeded for at least 25 days and were deemed to have successfully hatched at least one chick. Split according to sub‐population, 35 bird‐years were assigned to Wexford and 50 to Islay. The breakdown by sub‐population (given as, Wexford value | Islay value) was 9 (25.7%) | 10 (20.0%) bird‐years with no incubations, 21 (60.0%) | 24 (48.0%) bird‐years that failed during incubation and 5 (14.3%) | 16 (32.0%) bird‐years deemed to successfully have hatched at least one chick. The location of nest sites for each sub‐population and for individuals nesting in multiple years can be seen in Figures S1 and S2.

#### 3.1.3 Modelling variation in incubation initiation and success

Probability of incubation initiation was higher for the Islay sub‐population, but the parameter estimate (with Wexford as reference site) overlapped zero (*β* = 0.65 [CI: −0.78, 2.09]). All parameter estimates for environmental and phenological variables also overlapped zero, meaning there was no substantial support for any of the explanatory variables (Figure S8). Breeding success was also higher for the Islay sub‐population, but this parameter estimate marginally overlapped zero (*β* = 1.73 [CI: −0.03, 3.49]). All parameter estimates for environmental and phenological variables overlapped zero besides Greenland arrival date (*β* = −0.93 [CI: −0.21, −1.65]) (Figure S9). This suggests that birds arriving earlier on the breeding grounds have a higher likelihood of breeding successfully.

#### 3.1.4 Modelling variation in nest survival

**FIGURE 4:**
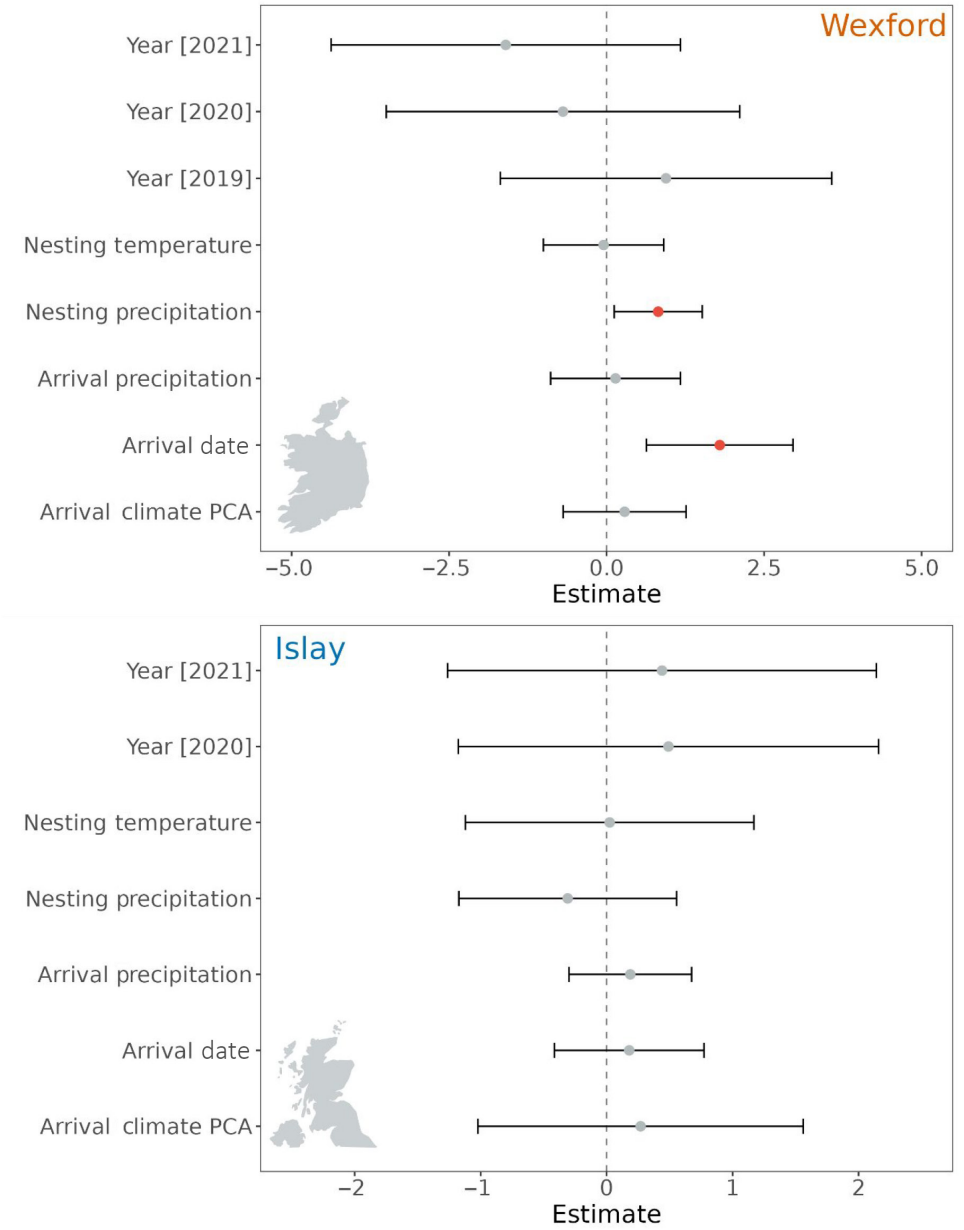
Forest plots from mixed effects Cox proportional hazards models assessing drivers of nest survival in GWfG. Models for the Wexford sub‐population (top) and Islay sub‐population (bottom) are depicted (Red parameter estimates = 95% confidence interval not spanning zero).

**FIGURE 5:**
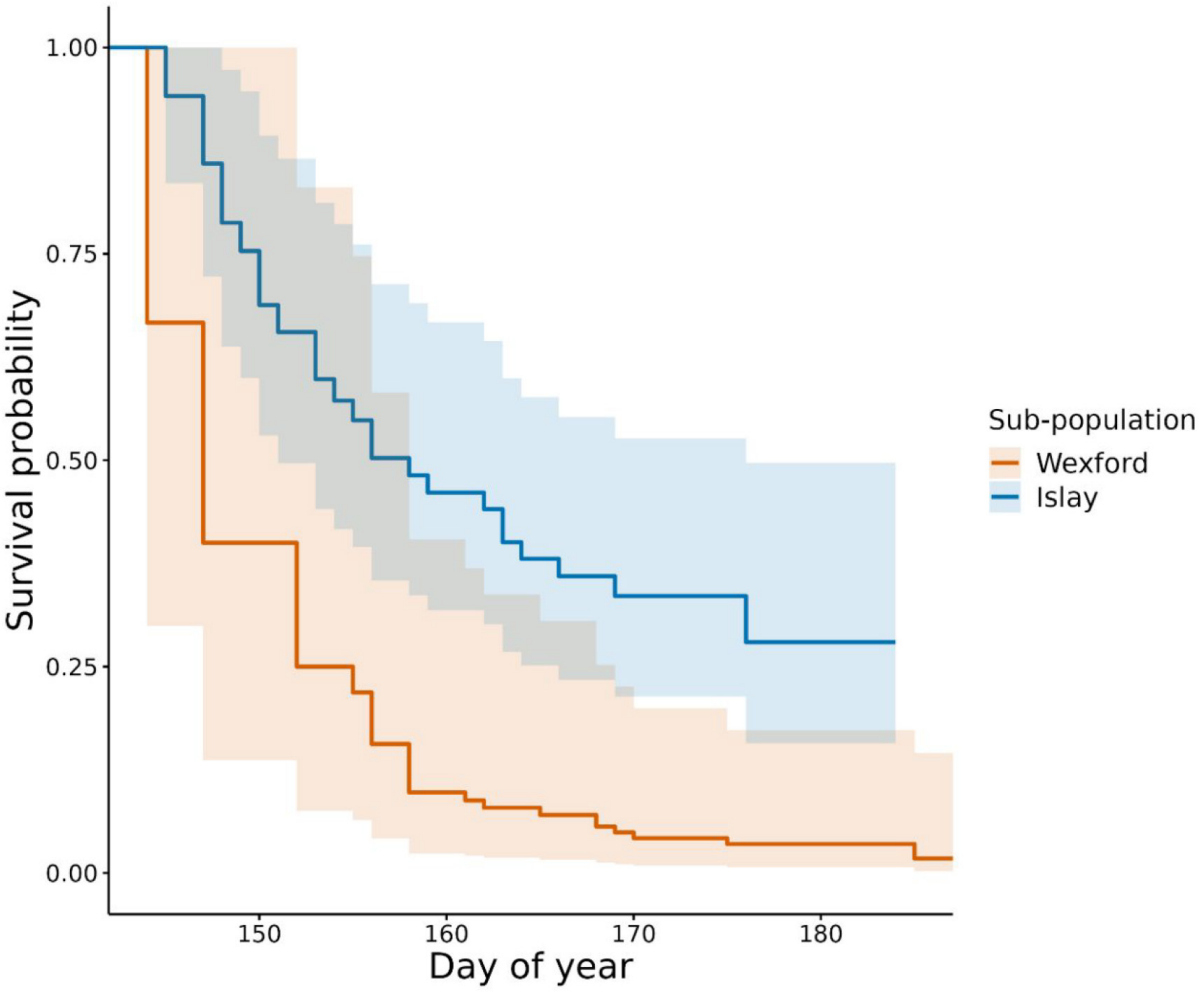
Comparison of nest survival for the Islay and Wexford sub‐populations from a mixed effects Cox proportional hazards model (95% confidence interval shaded).

**FIGURE 6:**
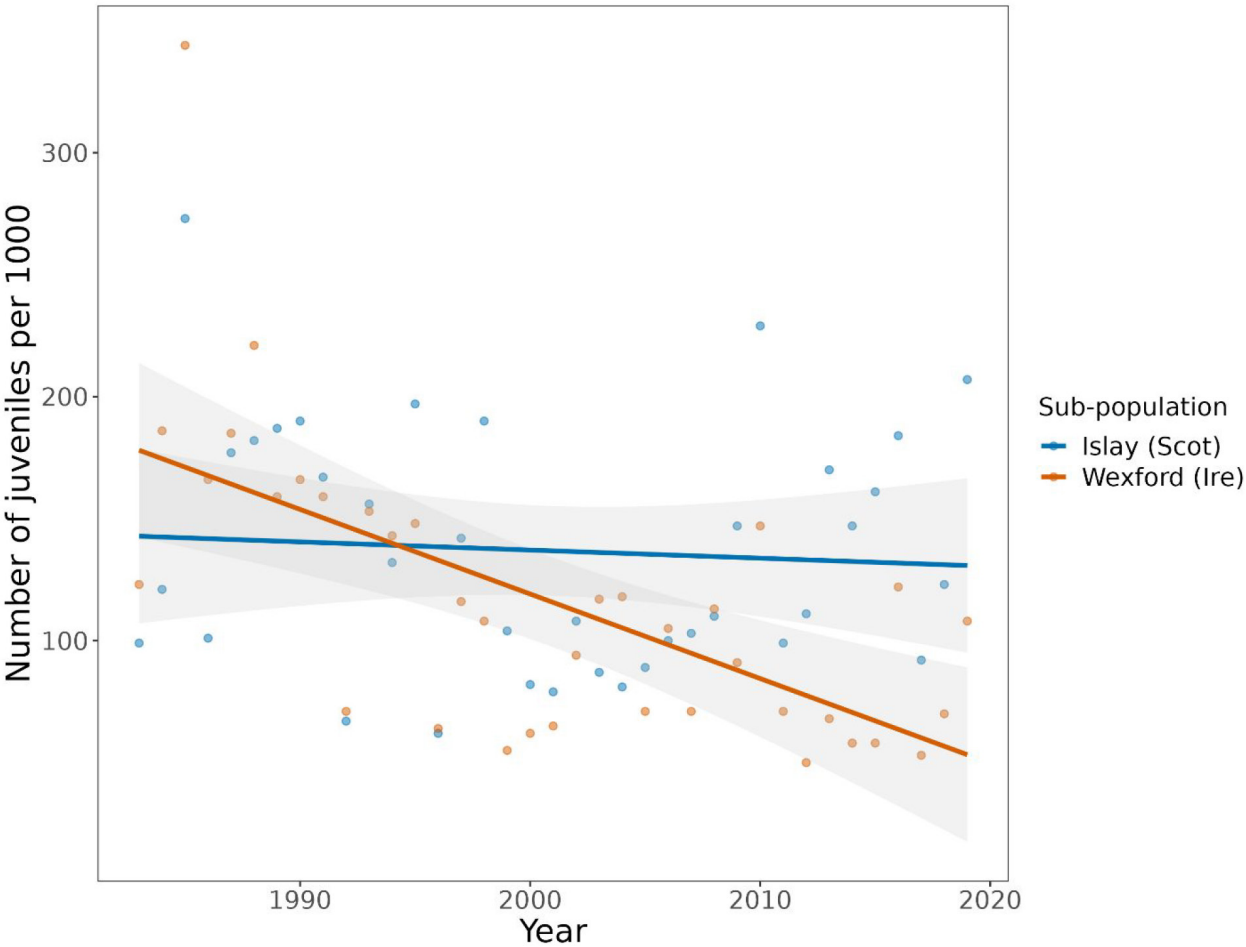
Temporal trend in the number of juveniles per 1000 birds in two sub‐populations of GWfG; Islay (blue) and Wexford (orange) (95% confidence intervals of slope estimates shaded grey).

We first ranked six different versions of the global model (Equation 7) with AICc, these versions differed by allowing the time‐varying environmental variables, ${\text{Temp}}_{\mathrm{iy}}\left(t\right)$ and ${\mathrm{Pr}}_{\mathrm{iy}}\left(t\right)$, to capture the climate over window lengths of 1, 2, 3, 4, 5 and 10 days. The model with the lowest AICc for the Islay sub‐population contained the 10‐day window (Table S2), but the 95% confidence interval for every parameter estimate overlapped zero (Figure 4), suggesting there was little support for environmental or phenological factors influencing nest survival. The model with the lowest AICc for the Wexford sub‐population contained a 4‐day window of temperature and precipitation (Table S2). The 95% confidence interval for two parameter estimates did not overlap zero (Figure 4), Greenland arrival date (*β* = 2.72 [CI: 0.99, 4.46]) and time‐dependent precipitation calculated over a 4‐day rolling window (*β* = 0.84 [CI: 0.14, 1.53]). These results suggest a significantly higher risk of nest failure for individuals that arrived later in Greenland and were exposed to more precipitation during incubation. We also compared nest survival between the sub‐populations and found that the risk of nest failure was lower for the Islay sub‐population (Figure 6) (*β* = −1.13 [CI: −1.89, −0.38]), and the 95% confidence interval did not overlap zero (Figure 5).

### 3.2 Population‐level results

#### 3.2.1 Temporal trends in productivity

We found a significant negative temporal trend in productivity for the Wexford sub‐population (*β* = −3.47 [CI: −5.14, −1.79]) but not for the Islay sub‐population (*β* = −0.33 [CI: −2.01, 1.34]), and there was a significant difference between these slope estimates (*β* = 3.13 [CI: 0.77, 5.50]) (Figure 6). A slope estimate of −3.47 for the Wexford sub‐population is equivalent to a decline of 3.47 juveniles per 1000 birds every year and equates to a reduction of 128.2 juveniles per 1000 birds over the 37‐year study period.

#### 3.2.2 Modelling variation in productivity

For the Islay sub‐population, the top ranked model explaining variation in productivity included environmental conditions calculated over the entire breeding period, whereas for the Wexford sub‐population it included environmental conditions during the pre‐hatching period (Table 1). Notably there was weak support for the effect of post‐hatch period environmental conditions on productivity in both sub‐populations. The parameter estimates from the top ranked models are presented in Table 2.

**TABLE 2:** Parameter estimates from the top models presented in Table 1, to understand variation in productivity of the Islay and Wexford sub‐populations.

Response variable	Explanatory variable	*β* [95% CI]
*Juv*_*Wexford*_	*Intercept*	510.21 [398.76, 621.67]
	*Freeze*	−5.99 [−7.35, −4.62]
	*Pr*	−1.22 [−2.43, −0.004]
	*Tr*_*Dec*_	−324.99 [−458.93, −191.05]
	*Freeze*: *Tr*_*Dec*_	4.11 [2.32, 5.89]
	*Pr*: *Tr*_*Dec*_	1.70 [0.15, 3.25]
*Juv*_*Islay*_	*Intercept*	516.03 [376.75, 655.31]
	*Freeze*	−5.69 [−7.50, −3.87]
	*Pr*	−0.95 [−2.34, 0.45]
	*Tr*_*Dec*_	−261.24 [−437.67, −84.81]
	*Freeze*: *Tr*_*Dec*_	3.92 [1.56, 6.28]
	*Pr*: *Tr*_*Dec*_	0.08 [−1.83, 1.99]

*Note*: For Wexford this was *Freeze* and *Precip* during the pre‐hatching period and for Islay during the entire breeding season.

**FIGURE 7:**
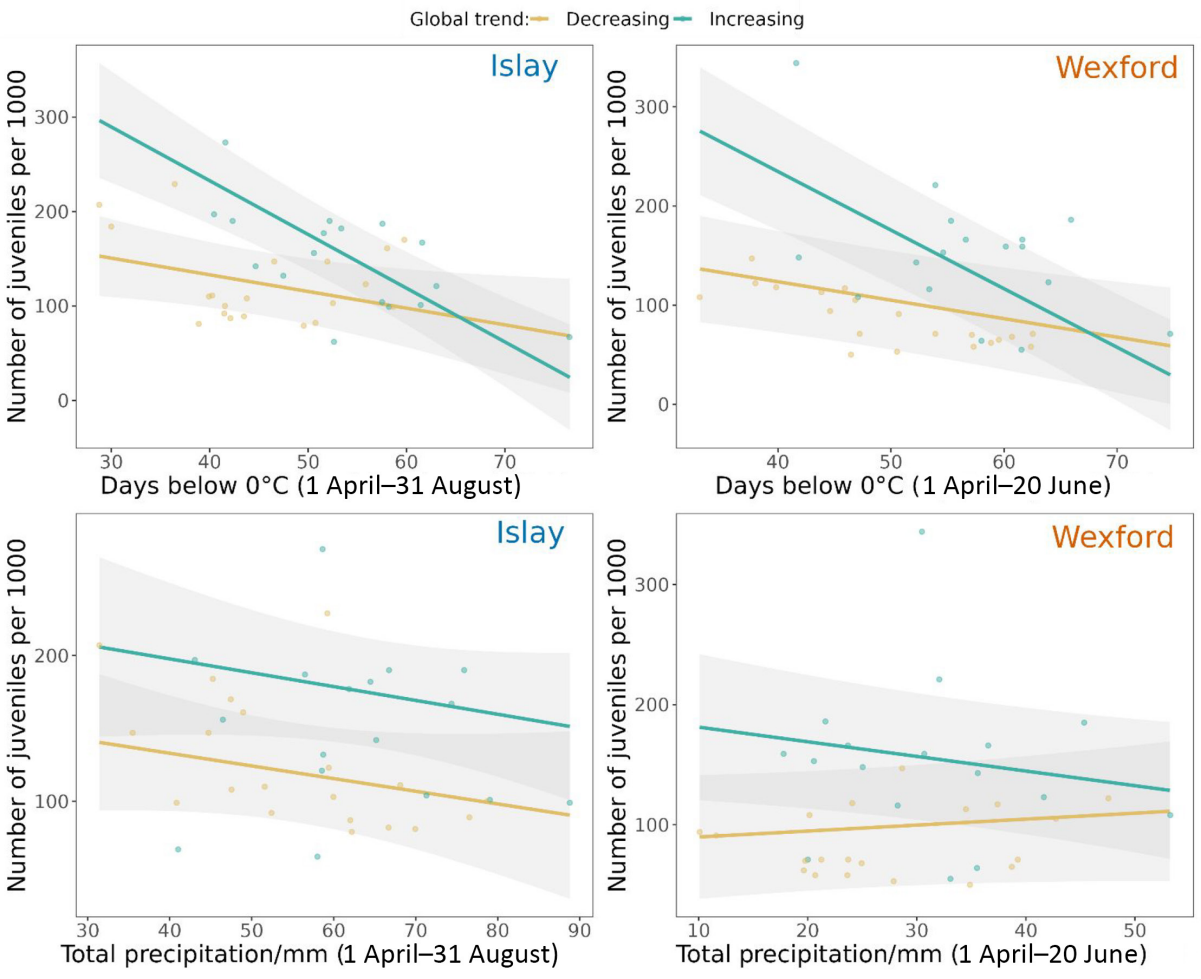
Relationships between the number of juvenile GWfG per 1000 birds and climatic variables. The top row depicts the relationships with the ‘number of days below 0°C’ and the bottom row depicts the relationship with ‘total precipitation’. For the Islay sub‐population (left column) climatic variables are calculated over the entire breeding period (1 April–31 August) and for the Wexford sub‐population over the pre‐hatching period (1 April–20 June). 95% confidence intervals of slopes estimates are shaded grey.

For the Islay sub‐population (Figure 7), there was a negative relationship between the ‘number of days below 0°C’ during the entire breeding season (1 April–31 August) and productivity during the metapopulation increase (*β* = −5.74 [CI: −7.56, −3.91]) and decrease (*β* = −1.79 [CI: −3.32, −0.26]). The slope of the relationship was significantly steeper during the metapopulation increase (*β* = 3.94 [CI: 6.31, 1.57]). The relationship between ‘total precipitation’ during the entire breeding season and productivity was non‐significant during both the metapopulation increase (*β* = −0.21 [CI: −0.55, 0.13]) and decrease (*β* = −0.24 [CI: −0.61, 0.12]). There was a significant decrease in the ‘number of days below 0°C’ (*β* = −0.39 [CI: −0.65, −0.14]) and the ‘total precipitation’ (*β* = −0.59 [CI: −1.06, −0.13]) during the entire breeding season for the Islay sub‐population between 1983 and 2019 (Figure S10). The estimate of phi, autocorrelation parameter measuring strength of association between successive productivity values, was 0.52.

For the Wexford sub‐population (Figure 7) there was a negative relationship between the ‘number days below 0°C’ during the pre‐hatching period (1 April–20 June) and productivity during both the metapopulation increase (*β* = −5.99 [CI: −7.35, −4.62]) and decrease (*β* = −1.87 [CI: −3.12, −0.61]). The slope was significantly steeper during the metapopulation increase (*β* = 2.93 [CI: 4.75, 1.11]). There was a significant negative relationship between ‘total precipitation’ during the pre‐hatching period and productivity during the metapopulation increase (*β* = −0.30 [CI: −0.60, −0.0001]) but not during the metapopulation decrease (*β* = 0.12 [CI: −0.11, 0.36]), and there was a significant difference between the two slope estimates (*β* = 0.43 [CI: 0.04, 0.81]). There was a significant decrease in the ‘number of days below 0°C’ (*β* = −0.32 [CI: −0.57, −0.07]) but no change in the ‘total precipitation’ (*β* = −0.13 [CI: −0.40, 0.14]) during the pre‐hatching period for the Wexford sub‐population between 1983 and 2019 (Figure S10). The estimate of phi, autocorrelation parameter, was 0.79.

## 4 DISCUSSION

The population‐ and individual‐level analyses presented here demonstrate important differences in the demography of two sub‐populations and how they relate to climatic conditions. At the population‐level it appears that correlations between productivity and temperature/precipitation weakened between the periods of metapopulation increase (pre‐1999) and decrease (post‐1999) (precipitation effect—Wexford only, Figure 7). The weakening of these correlations coincides with a significant decline in productivity for the Wexford but not the Islay sub‐population (Figure 6). At the individual‐level, during 2018–2021, birds from the Wexford sub‐population had lower nest survival than those from the Islay sub‐population (Figure 5). Moreover, earlier arrival dates and lower rates of precipitation during incubation increased nest survival for the Wexford but not the Islay sub‐population. The factor(s) limiting productivity in the Wexford sub‐population appear to cause low nest survival and likely differ to the limiting factors in the Islay sub‐population. Thus, we show that even within a metapopulation with moderate exchange and relatively small spatial separation, relationships between demography and the environment can vary markedly across sub‐units.

### 4.1 Integrating population‐level and individual‐level results

Productivity of both sub‐populations pre‐1999 (metapopulation increase) was negatively correlated with the number of days below 0°C and total precipitation (Wexford only). There are similar findings in other Arctic breeding wildfowl (Doyle et al., 2020; Jensen et al., 2014; Nolet et al., 2020) where warmer temperatures and less precipitation, which often falls as snow, promotes earlier access to more abundant food (Anderson et al., 2014) and nest sites (Wisz et al., 2008). Reproductive success subsequently increases as individuals incubate earlier and in better body condition (Anderson et al., 2014; Jensen et al., 2014). Warmer breeding seasons also lengthen resource abundance peaks which increases chick growth rates and survival (Lameris et al., 2022). We perhaps only see a precipitation effect in the Wexford sub‐population due to colder, more northerly breeding sites where early precipitation is likely to be snow, and consequently delay resource access.

Pre‐1999 (metapopulation increase) reproductive success of the two sub‐populations was most strongly correlated with climatic conditions during different breeding stages, pre‐hatching for Wexford and the entire breeding season for Islay. Early breeding season snow cover is highly influential on productivity in other high Arctic nesting geese, for example Pink‐footed Geese, *Anser brachyrhynchus* (Jensen et al., 2014). Therefore the difference could reflect greater constraints for the Wexford sub‐population during the pre‐hatching period due to later snow melts and resource access in their more northerly breeding grounds (Nolet et al., 2020). For the Islay sub‐population, snow melt may generally not be late enough to have catastrophic implications for breeding. After 1999 (metapopulation decrease) the correlation between productivity and environmental variables weakened, perhaps suggesting that different factor(s) were more strongly driving productivity. This is partly corroborated by our individual‐level result that climatic variables did not explain variation in incubation propensity or nest survival for the Islay sub‐population (although precipitation during incubation may still be an important driver for the Wexford sub‐population).

At the individual‐level the decline in productivity of the Wexford sub‐population is most likely due to low nest survival (although we did not measure chick survival). Since arrival date and precipitation during incubation altered nest survival in the Wexford sub‐population but no environmental or phenological factors were important for the Islay sub‐population, it seems likely that the factors limiting reproductive success in the two sub‐populations differ. This suggests that either breeding ground abiotic/biotic factors that limit breeding success are having greater impacts in the north of the breeding range or a constraint earlier in the annual cycle, where the sub‐populations use broadly different areas, could be having negative carry‐over effects on reproductive success for the Wexford sub‐population (Inger et al., 2010; Norris et al., 2004). However, carry‐over effects are less plausible since the fat and mass accumulation of Wexford birds staging in Iceland was unchanged between 1998 and 2007 (Fox et al., 2012). For the Islay sub‐population, it is unclear what factors currently drive nest survival. An explanation for our results is that a warming Islay breeding range (Figure S10) is increasing access to early season resources, allowing more Islay individuals to breed and in better body condition (Dickey et al., 2008; Nolet et al., 2020), independent of arrival time. Therefore there is no significant relationship between climatic and/or phenological factors and nest survival as resources are abundant.

The two drivers of nest survival in the Wexford sub‐population (arrival date and precipitation, Figure 4) could indicate the cause(s) of low breeding success and disparity between sub‐populations. Earlier breeding ground arrival is widely linked to increased breeding success as early individuals occupy higher quality feeding/nesting sites and can be of higher innate quality (Mckellar et al., 2013; Morrison et al., 2019). In the more northerly Wexford breeding range fewer snow‐free sites are available upon arrival, which can increase reliance on capital resources for egg production (Sharp et al., 2013) (a breeding strategy that utilizes endogenous stores from earlier migratory stages; Inger et al., 2010). Therefore, early arriving Wexford birds can monopolise the few available feeding sites and initiate incubation in better condition and increase breeding success (Dickey et al., 2008). In comparison, the Islay breeding range has more abundant resources and therefore early arrival confers no real advantage. Alternatively, since, breeding ground arrival date was moderately repeatable within individuals. This may reflect intrinsic differences in migratory timings that have arisen due to variation individual quality, for example migration speed (Both & Visser, 2001). If higher individual quality is also linked to higher capital resource investment, then we would expect a stronger effect of arrival date on breeding success in Wexford birds as they require more capital resource investment for breeding as food availability prior to nesting is sparser (Sharp et al., 2013). Another potential contributing factor is the recently colonized Canada goose, *Branta canadensis interior*, population (Fox & Glahder, 2010). Canada geese could compete with GWfG but further research is required to understand their distribution in west Greenland and whether their occurrence is more detrimental further north where early breeding season resources are already more limited.

More precipitation, over a 4‐day window, increased nest failure in the Wexford sub‐population. Since average temperatures are below 0°C during incubation (−0.44 ± 0.95°C), this precipitation is likely snow. The same relationship has been found in Red‐breasted Geese, *Branta ruficollis*, and Barnacle Geese, *Branta leucopsis* (Doyle et al., 2020; Kostin & Mooij, 1995), and is caused by increased snow cover limiting access to food and/or precipitation chilling eggs leading to incubation failure. Since total precipitation during the breeding season has not increased since 1999 and was not correlated with population‐level breeding success (Figure S10), it is unlikely the direct effects of precipitation have caused declining breeding success in the Wexford sub‐population. Instead factors that mediate the effect of precipitation on breeding could have altered, enabling easier detection of the influence of precipitation on breeding success at the individual‐level. For instance, poor body condition due to a carry‐over effect could lengthen nest recesses (Tombre et al., 2012) and increase the likelihood of precipitation chilling eggs. Alternatively, increased predator abundance could make precipitation effects easier to detect. Snow cover can increase nest recess duration as suitable feeding/drinking sites are further from nests, for example (Guerena et al., 2016; Juhasz et al., 2020), making nest detection by predators more efficient (Lecomte et al., 2009).

## 5 CONCLUSIONS

In the two largest GWfG sub‐populations the correlation between breeding ground climatic conditions and reproductive success have weakened. This has coincided with different temporal trends in reproductive success. For the Wexford sub‐population, reproductive success has declined and appears to be caused by low nest survival. Drivers of low nest survival could be poor pre‐breeding body condition, limited breeding ground resource availability and/or nest predation but these factors remain untested. Our multi‐level approach allows mechanistic drivers of population decline to be examined, in more correlative studies, mechanisms are harder to determine. If one were to amalgamate demographic data across both sub‐population then it could give misleading results as to how differences in individual‐level reproductive success contribute to changes in productivity at the metapopulation‐level. Identifying distinct sub‐populations within metapopulations that experience different environmental conditions is vital for decomposing demographic parameters and identifying where and when drivers of population decline act (Morrison et al., 2016).

### 5.1 Future research and conservation

Our findings highlight that sub‐populations occupying adjacent, overlapping regions can differ in their demography and the climatic drivers. Our findings have the following implications for metapopulation monitoring and conservation:

1. Studies monitoring a single sub‐population and presuming demographic rates are representative potential risk missing important sub‐population‐level trends and dynamics.
2. Conservation practitioners managing multiple sub‐populations may require bespoke conservation strategies for each that target different proximate mechanism(s) of population change.
3. Studying the demography of sub‐populations could allow practitioners to pinpoint those that will benefit most from conservation intervention and where benefits may spread to connected sub‐populations via movement (Hanski, 1998); maximising the effectiveness of limited management resources (McDonald‐Madden et al., 2008).

## AUTHOR CONTRIBUTIONS

**Luke Ozsanlav‐Harris:** Conceptualization (equal); data curation (equal); formal analysis (equal); methodology (equal); project administration (equal); resources (equal); visualization (equal); writing – original draft (equal); writing – review and editing (equal). **Stuart Bearhop:** Conceptualization (equal); methodology (equal); project administration (equal); resources (equal); supervision (equal); writing – review and editing (equal). **Mitch D. Weegman:** Funding acquisition (equal); project administration (equal); resources (equal); writing – review and editing (equal). **Larry R. Griffin:** Conceptualization (equal); data curation (equal); funding acquisition (equal); project administration (equal); resources (equal); supervision (equal); writing – review and editing (equal). **Geoff M. Hilton:** Conceptualization (equal); funding acquisition (equal); investigation (equal); project administration (equal); resources (equal); supervision (equal); writing – review and editing (equal). **Lei Cao:** Funding acquisition (equal); project administration (equal); resources (equal); writing – review and editing (equal). **Alyn J. Walsh:** Data curation (equal); project administration (equal); resources (equal); writing – review and editing (equal).

## CONFLICT OF INTEREST STATEMENT

None to declare.

## Supporting information


Appendix S1

## Data Availability

The data and code at the time of publication can be found on Zenodo at the following link: https://zenodo.org/record/8116139. A working version of the data and code used in this manuscript are hosted in a GitHub repository here; https://github.com/LukeOzsanlav/GWfGMetapopDemography.
