# Supplementary material for: Differing drivers of decline within a migratory metapopulation has implications for future conservation
Source: Ecol Evol. 2023 Jul 14;13(7):e10281. doi: 10.1002/ece3.10281 (PMC10347676; doi:10.1002/ece3.10281)
Supplement: Supplementary file 1 — Appendix S1 [file ECE3-13-e10281-s001.docx]

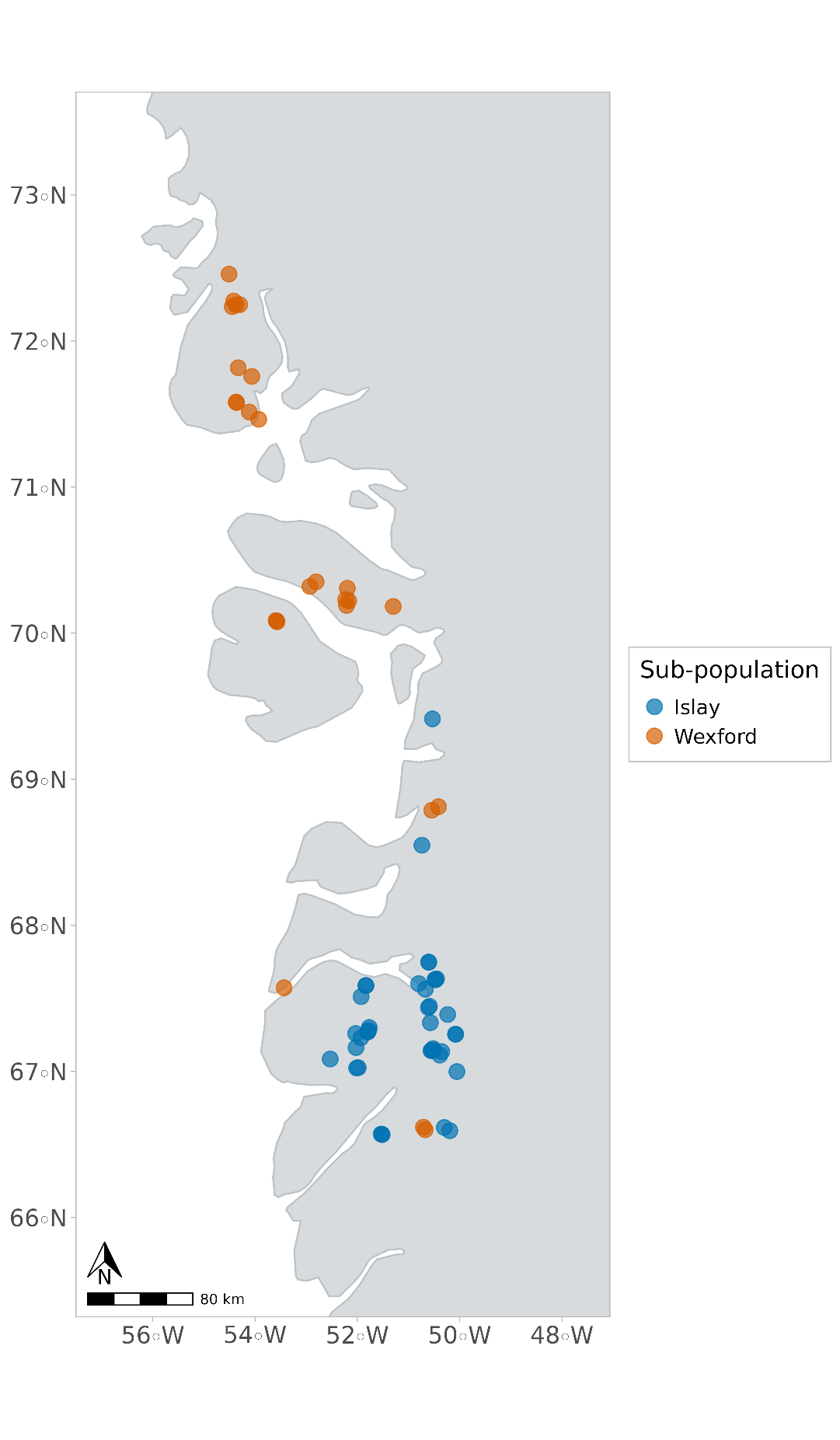
**Supporting Information 1**

**Supplementary figure 1.** All nest locations from two sub-populations of Greenland White-fronted Geese in West Greenland. Incubation events were identified for each tracked bird and the putative nest site then derived from the GPS data.


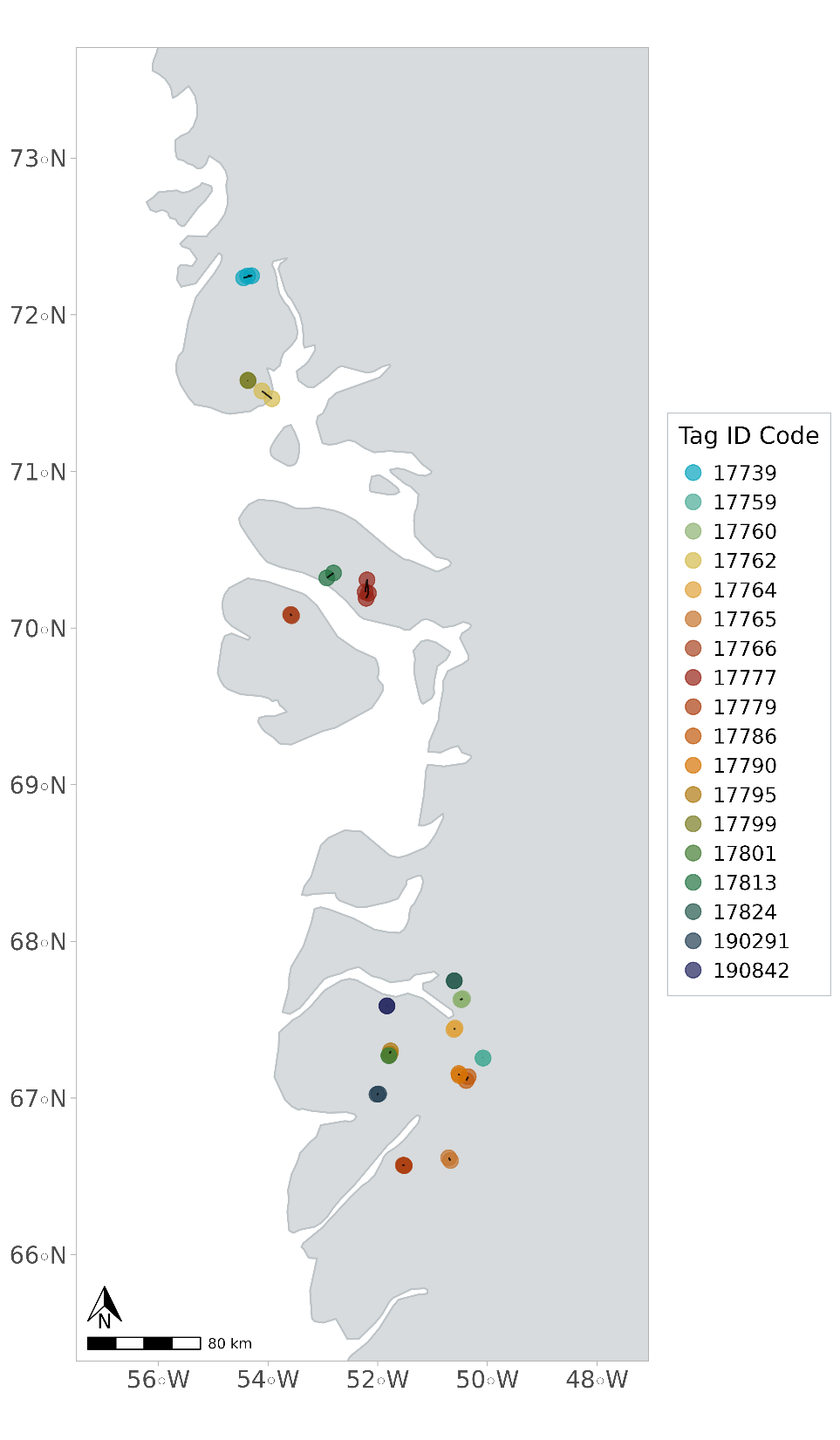


**Supplementary figure 2.** Repeat nest locations for individuals tracked and classified as breeders for more than two breeding seasons from Greenland White-fronted Geese in West Greenland. Incubation events were identified for each tracked bird and the putative nest site then derived from the GPS data. Small black lines connect the nest locations of the same individual and most individuals use very similar sites for nesting in successive years.

**
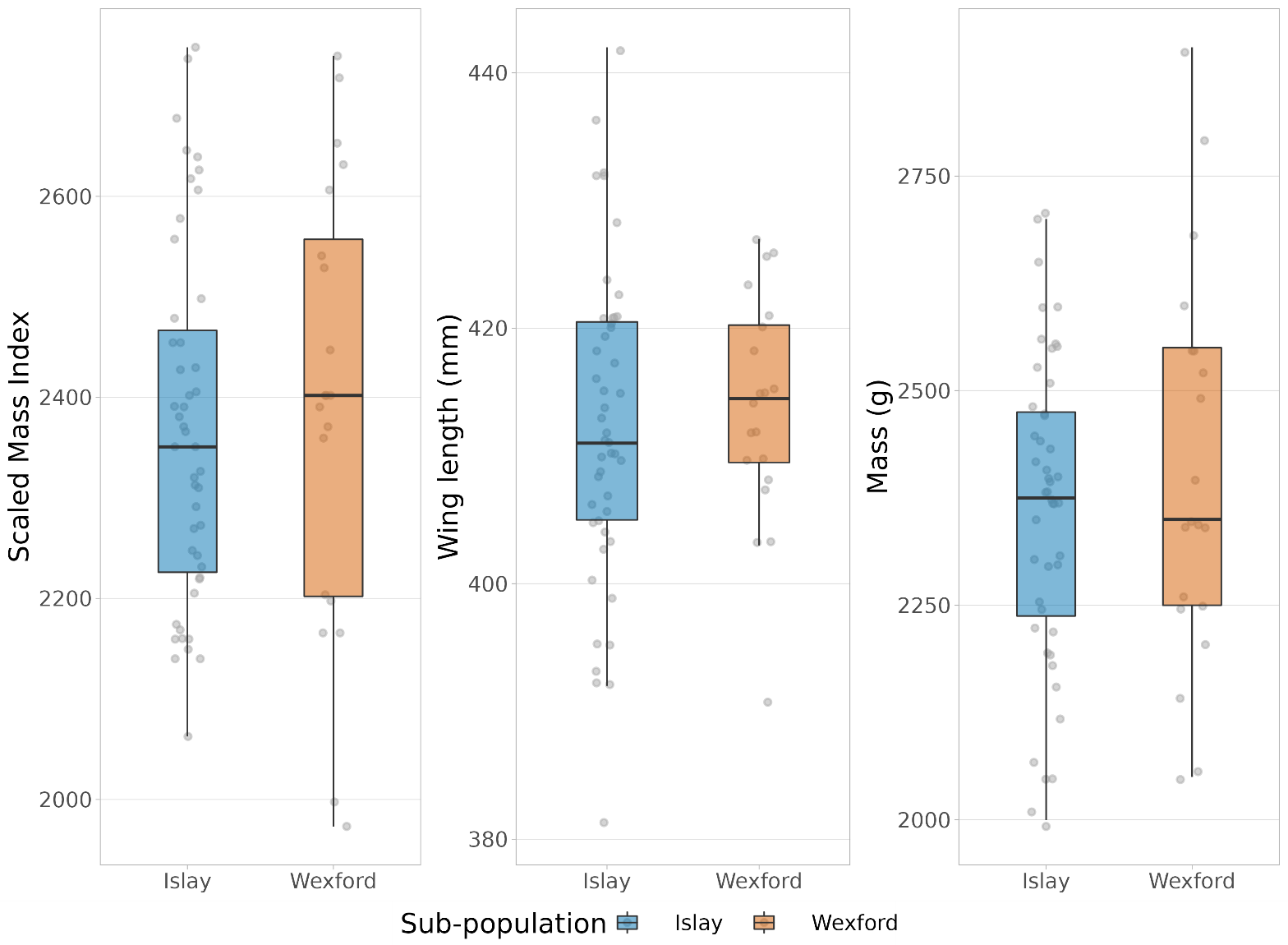
**

**Supplementary figure 3.** Morphological comparisons between two sub-populations of Greenland White-fronted geese caught during trapping for the deployment of biologging devices (n=67). Measurements were taken during the non-breeding period in the UK or late autumn staging period in Iceland. Morphological traits were compared using linear models with sub-population (two-level factor with Islay being the reference level) used as the sole explanatory variable. There was no significant differences for body mass in grams (β = 42.8 [CI: -61.9, 147.5]), wing length in millimetres (β = 1.5 [CI: -4.7, 7.7]) and scaled mass index (β = 25.2 [CI: -77.4, 127.8]) between sub-populations. Scaled mass index was calculated using body mass and head to bill length using the method in Peig and Green (2009)

**
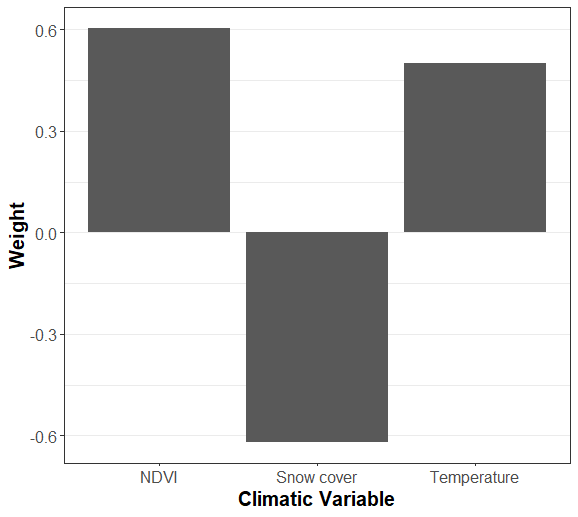
**

**Supplementary figure 4.** Loadings from the first axis of a PCA on three climatic variables experienced by individual GWfG during the 10 days after arriving on the breeding grounds. This axis explained 75.3% of the total variation.


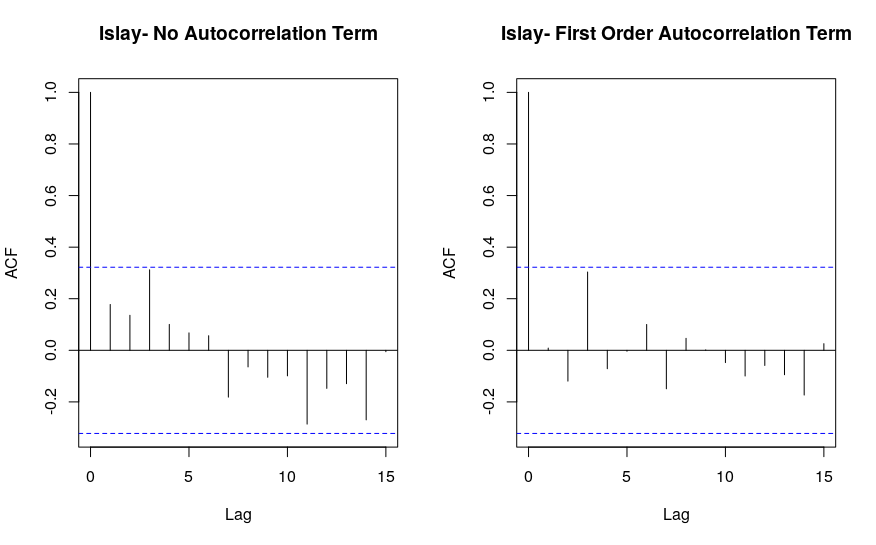


**Supplementary figure 5.** Autocorrelation plots of the residuals from a model examining the influence of environmental variables on the population-level productivity of the Islay sub-population of GWfG (equation 7). The environmental variables in these models are those from the model with the lowest AICc in Table 1 (number of days below 0°C and total precipitation over the whole breeding season). The plot on the left had no autocorrelation term and the plot on the right had first-order autoregressive covariance structure term with respect to year. When a second-order autoregressive term was used the AICc of the models increased and the autocorrelation plot looked similar to that presented here for a first-order model.


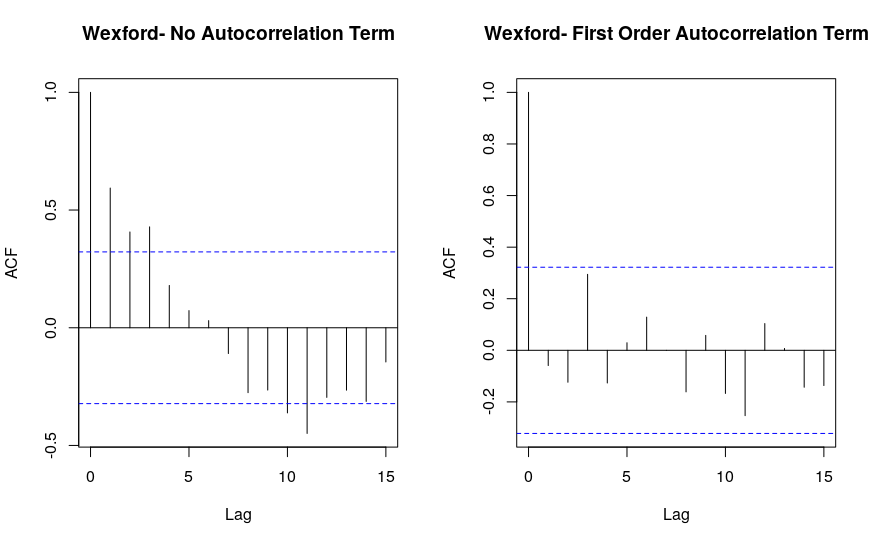


**Supplementary figure 6.** Autocorrelation plots of the residuals from a model examining the influence of environmental variables on the population-level productivity of the Wexford sub-population of GWfG (equation 7). The environmental variables in these models are those from the model with the lowest AICc in Table 1 (number of days below 0°C and total precipitation during the pre-hatching period of the breeding season). The plot on the left had no autocorrelation term and the plot on the right had first-order autoregressive covariance structure term with respect to year. When a second-order autoregressive term was used the AICc of the models increased and the autocorrelation plot looked similar to that presented here for a first-order model.


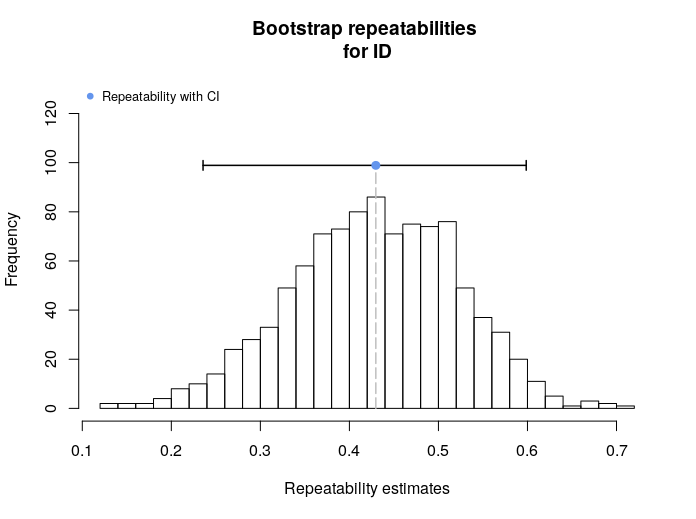


**Supplementary figure 7.** Repeatability of year centred breeding ground arrival dates for adult female Greenland White-fronted Geese. The histogram is the result of 1000 parametric bootstraps and the mean repeatability plus 95% confidence interval are plotted above.

**
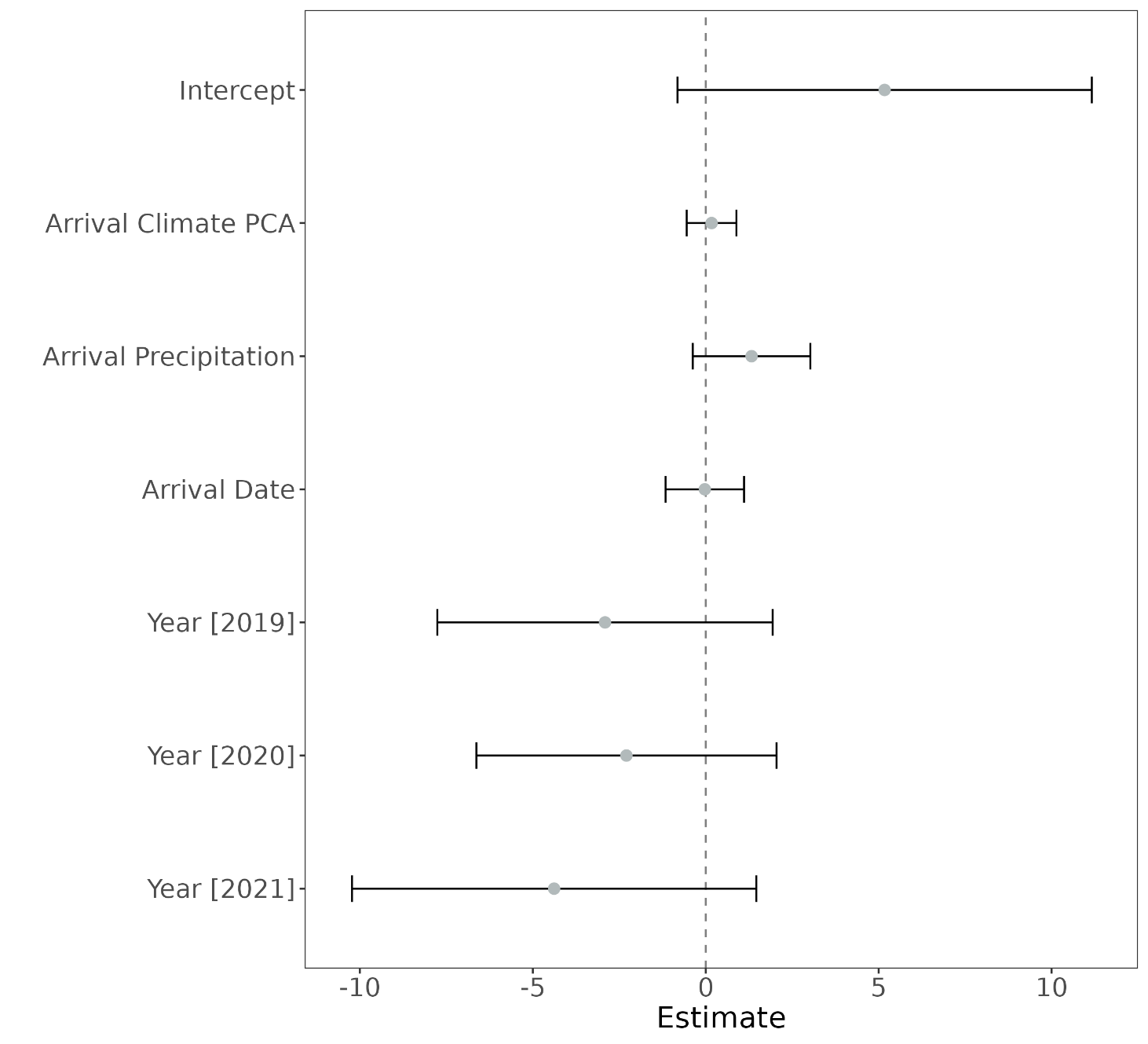
**

**Supplementary figure 8.** Forest plots from modelling differences in the Breeding deferral of Greenland White-fronted Geese using a mixed effects generalised liner model. Where the 95% confidence interval of the parameter estimate does not overlap with zero the mean estimate is red.

**
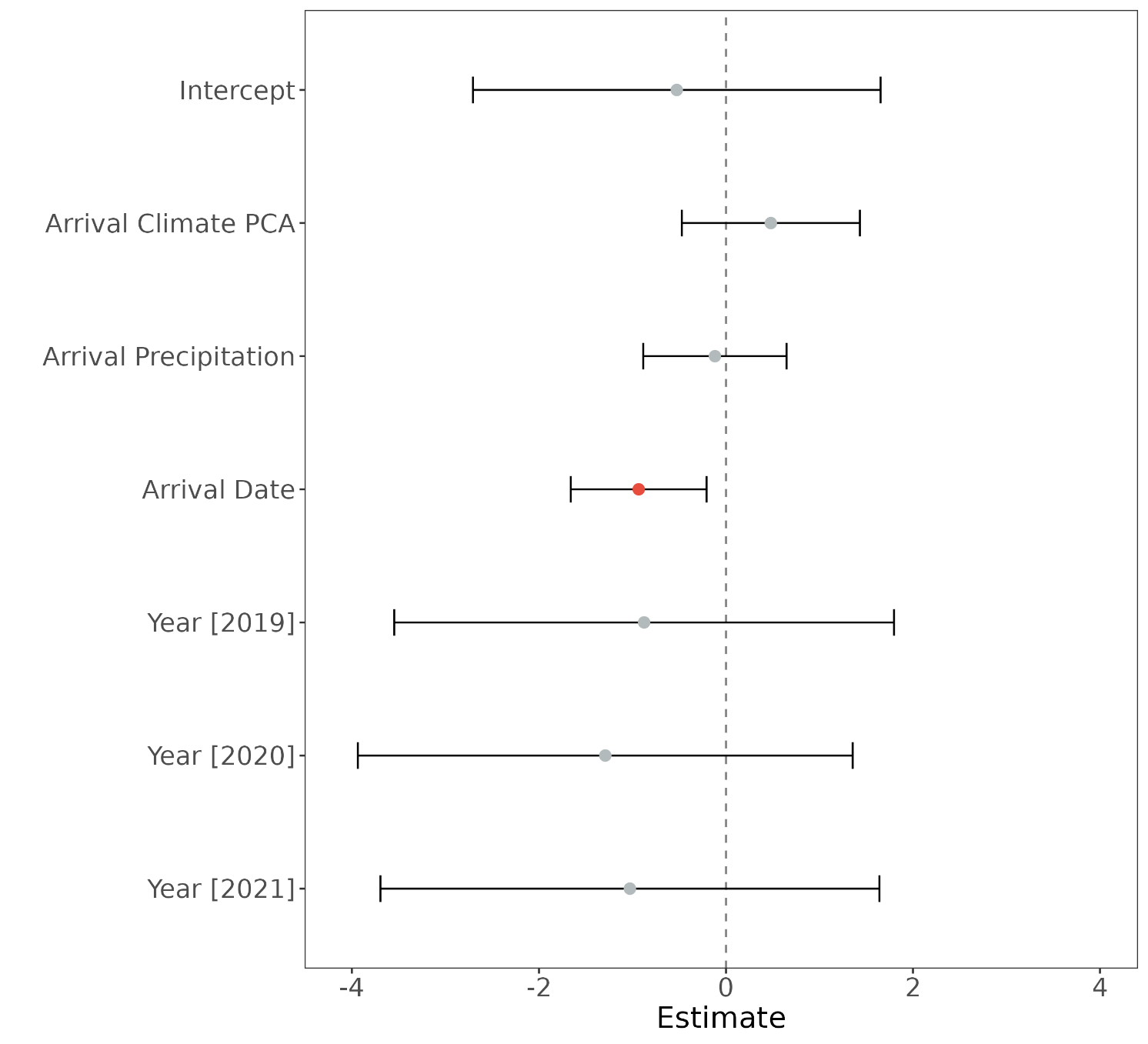
**

**Supplementary figure 9.** Forest plots from modelling differences in the breeding success of Greenland White-fronted Geese using a mixed effects generalised liner model. Where the 95% confidence interval of the parameter estimate does not overlap with zero the mean estimate is red.

**
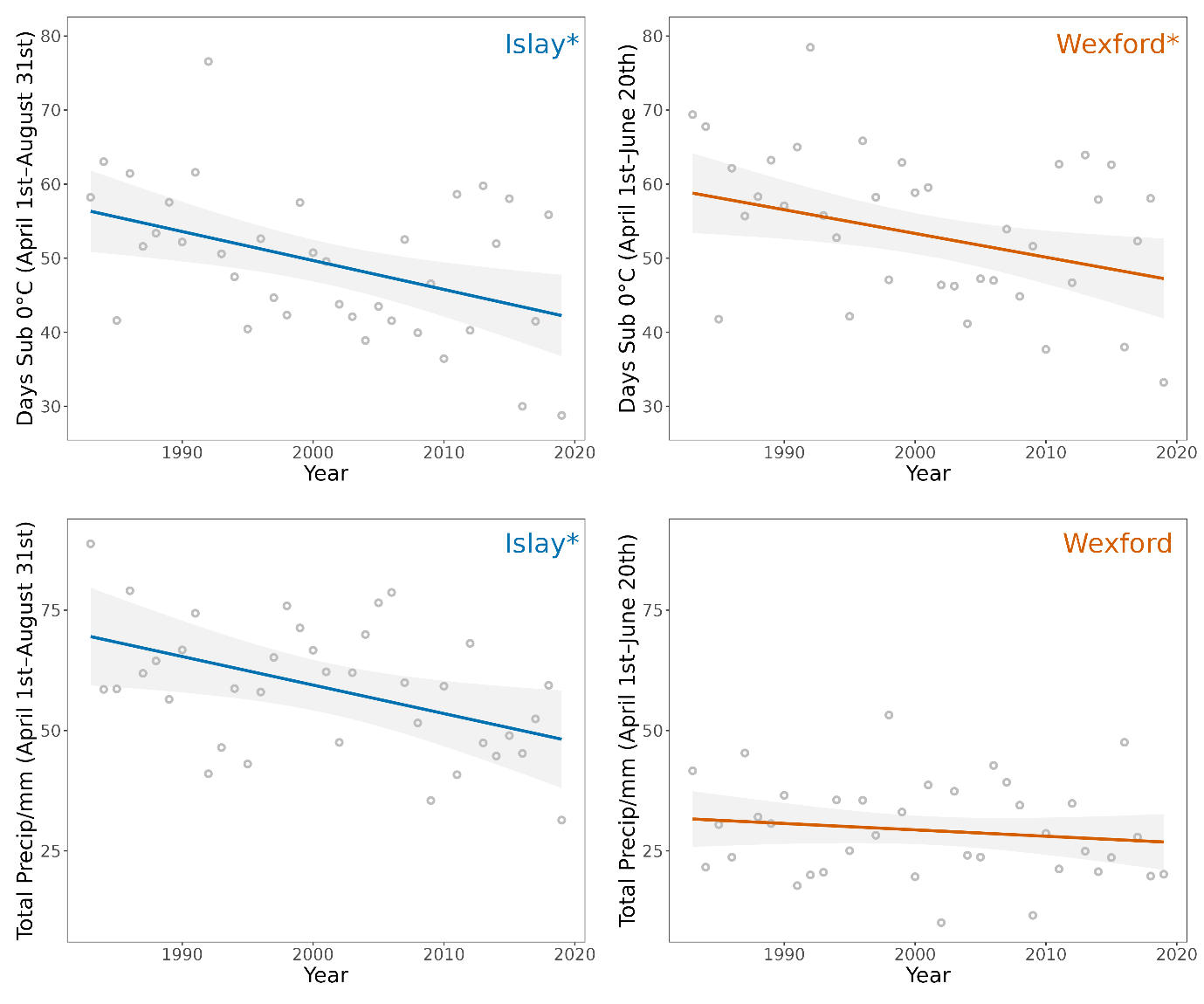
**

**Supplementary figure 10.** The temporal trends for climatic variables in the breeding ranges of two sub-populations of Greenland White-fronted Goose. Trends in the number of days below 0°C (top) and total precipitation (bottom) are depicted for the Islay (left) and Wexford (right) sub-populations. * indicates that the 95% confidence interval of the regression slope did not span zero. Note: climactic conditions are calculated over the entire breeding season for the Islay sub-population and the pre-hatching period for the Wexford sub-population.

**Supplementary Table 1.** Additional tags used to calculate Greenland arrival and estimate the repeatability of breeding ground arrival dates. These tags did not meet the data requirements to be used in the analysis to classify incubation. The additional tags used were Ecotone GPS-GSM device (Gdynia, Poland; c24g) and Ecotone ultrahigh frequency (UHF) device, (Gdynia, Poland; c24g). All tagging was performed under licence from the British Trust for Ornithology (permit no: /A/5436).

| **Tag Type** | **Ecotone GPS-GSM** | **Ecotone UHF** |
| --- | --- | --- |
| *Citation* | Gdynia, Poland | Gdynia, Poland |
| *Weight* | 24g | 24g |
| *Download* | 2G/3G network | Yagi Antennae |
| *GPS Fix Rate (mins)* | 30 | 15 (07:00-15:00 only) |
| *Sample Size* | 13 | 7 |

**Supplementary Table 2.** Comparing AICc values from nest survival models on two different sub-populations of Greenland White-fronted Geese. Multiple models were compared for each sub-population which differed in that fact that time-dependent environmental variables were averaged or summed for 1, 2, 3, 4, 5 or 10 days inclusively from the current day.

| **Sub-population** | **Window Size (days)** | **AICc** |
| --- | --- | --- |
| *Islay* | 10 | 155.97 |
| *Islay* | 1 | 156.42 |
| *Islay* | 5 | 156.53 |
| *Islay* | 4 | 157.12 |
| *Islay* | 2 | 157.77 |
| *Islay* | 3 | 157.77 |
| *Wexford* | 4 | 74.88 |
| *Wexford* | 3 | 75.70 |
| *Wexford* | 5 | 76.37 |
| *Wexford* | 1 | 76.85 |
| *Wexford* | 2 | 77.34 |
| *Wexford* | 10 | 78.37 |
